# Medical Subject Heading (MeSH) annotations illuminate maize genetics and evolution

**DOI:** 10.1186/s13007-017-0159-5

**Published:** 2017-02-23

**Authors:** Timothy M. Beissinger, Gota Morota

**Affiliations:** 1 USDA-ARS Plant Genetics Research Unit, Division of Plant Sciences, Division of Biological Sciences, MU Informatics Institute, University of Missouri, Columbia, MO 65211 USA; 2 Department of Animal Science, University of Nebraska, Lincoln, NE 68583 USA

**Keywords:** MeSH, Maize, Gene ontology (GO), Overrepresentation analysis (ORA), Domestication, Ear number, Seed size, Inflorescence

## Abstract

**Background:**

High-density marker panels and/or whole-genome sequencing, coupled with advanced phenotyping pipelines and sophisticated statistical methods, have dramatically increased our ability to generate lists of candidate genes or regions that are putatively associated with phenotypes or processes of interest. However, the speed with which we can validate genes, or even make reasonable biological interpretations about the principles underlying them, has not kept pace. A promising approach that runs parallel to explicitly validating individual genes is analyzing a set of genes together and assessing the biological similarities among them. This is often achieved via gene ontology analysis, a powerful tool that involves evaluating publicly available gene annotations. However, additional resources such as Medical Subject Headings (MeSH) can also be used to evaluate sets of genes to make biological interpretations.

**Results:**

In this manuscript, we describe utilizing MeSH terms to make biological interpretations in maize. MeSH terms are assigned to PubMed-indexed manuscripts by the National Library of Medicine, and can be directly mapped to genes to develop gene annotations. Once mapped, these terms can be evaluated for enrichment in sets of genes or similarity between gene sets to provide biological insights. Here, we implement MeSH analyses in five maize datasets to demonstrate how MeSH can be leveraged by the maize and broader crop-genomics community.

**Conclusions:**

We demonstrate that MeSH terms can be effectively leveraged to generate hypotheses and make biological interpretations in maize, and we provide a pipeline that enables the use of MeSH terms in other plant species.

**Electronic supplementary material:**

The online version of this article (doi:10.1186/s13007-017-0159-5) contains supplementary material, which is available to authorized users.

## Background

Technological advances in sequencing and phenotyping have accelerated in recent decades, enabling high-throughput studies aimed at associating genotypes and phenotypes. In many cases, the speed at which we can generate large sets of candidate associations from genome-wide association studies (GWAS) [1], selection mapping [2], and other approaches has surpassed our ability to draw meaningful biological conclusions from these candidates. However, as was recently described by Rausher and Delph [3], gene-identification is not always necessary to draw meaningful insights. Alternatively, it is often possible to look for recurrent patterns among distinct sets of candidate genes or regions in order to elucidate meaning. Annotation-based tests for enrichment or similarity represent one avenue for unraveling meaning from sets of candidates. In brief, these approaches involve identifying statistically enriched annotation terms among a list of candidate sites (usually genes or regions), or looking for similarity between terms corresponding to two sets of candidate sites, and inferring that there may be a biological explanation for the enriched or similar terms.

Commonly applied techniques often utilize gene ontology (GO) annotations [4], which provide putative descriptions of gene function [5, 6]. GO annotations are an important genomic tool to provide insight into biological interpretations of gene sets. However, despite their well-proven utility, there is growing interest in additional annotation-based approaches that can be leveraged to complement, support, enhance, or add to the patterns identified by GO. Included among this assortment of strategies are KEGG annotations [7], Disease Ontology [8], and Medical Subject Headings (MeSH), which were introduced at the National Library of Medicine (NLM) more than 50 years ago [9].

MeSH terms are the NLM’s controlled terminology, primarily used to organize and index information and manuscripts found in common databases such as PubMed [10]. By mapping from MeSH terms to manuscripts, and then to a list of candidate genes, a semantic pattern search for biological meaning can be conducted [11]. Recently, the MeSH Over-representation Analysis (ORA) Framework, a suite of software for conducting MeSH enrichment analyses using R [12] and Bioconductor [13], was developed [14]. MeSH analysis has proven useful for deducing meaning from sets of genes implicated across several agricultural animal species including in cattle, swine, horse and chicken [15, 16]. Here, we implement five MeSH analyses in maize, which collectively demonstrate how MeSH can been used to enrich biological understanding in crop species.

In this study, which is meant to be both a primer for MeSH-based analysis in maize and other crop plants, as well as an investigation of patterns that can be deduced regarding maize genetics and evolution, we identify over-represented MeSH terms among candidate genes identified from five distinct maize datasets: (1) regions under selection during maize domestication [17]; (2) regions under selection during maize improvement [17]; (3) regions under selection for seed size [18]; (4) regions under selection for ear number [19]; and (5) regions contributing to inflorescence traits [20]. After identifying significant MeSH terms, we also assess and test for semantic similarity, or MeSH-based relatedness, among the genes identified in each of these datasets to identify relationships among the genetic underpinnings of these traits/selection regimes.

## Methods

### Code availability

To enable implementation of MeSH analyses by other researchers, all scripts used in this study are available as annotated additional files in R-markdown format (Additional files 1, 2, 3, 4, 5, 6, 7). Scripts were written in R [12] and utilize Bioconductor [13], the MeSH ORA Framework including the “meshr” for ORA and the “Mesh.Zma.e.g.db” maize-specific mapping table [14], and MeSHSim [21]. The mapping table provides the necessary link between NCBI Entrez Gene IDs and NLM MeSH IDs. For maize, the mapping table was provided by gene2pubmed [22] with data licensed by PubMed. The GOstats R package [23] was used to implement GO ORA to generate a baseline that MeSH results could be compared to. Genome data was downloaded using the biomaRt R package [24]. Full analysis details are included within the reproducible scripts (Additional files 1, 2, 3, 4, 5, 6, 7).

### Datasets

We analyzed five publicly available datasets to identify enriched MeSH terms and look for semantic similarity between different traits and selection regimes. The datasets analyzed are described in Table 1. For the four datasets that involved contiguous regions (Domestication, improvement, seed size, and ear number), all genes that fell within the implicated regions were used for MeSH analysis. For the remaining dataset (inflorescence traits), which involved isolated SNPs identified through GWAS instead of genomic regions, all genes within 10 kb of the implicated SNPs were used for MeSH analysis. All gene models and gene locations were based on the maize reference genome version 2 [25].

**Table 1:** Datasets used in this study, including reference information where full details can be found and a brief description of each

Dataset	Reference	Description
Domestication	Hufford et al. [17]	Regions selected during domestication from teosinte to maize
Improvement	Hufford et al. [17]	Regions selected during post-domestication maize improvement
Seed size	Hirsch et al. [18]	Regions artificially selected for seed size in a long-term selection experiment
Ear number	Beissinger et al. [19]	Regions artificially selected for ear number in a long-term selection experiment
Inflorescence traits	Brown et al. [20]	SNPs associated with inflorescence traits from a genome-wide association study

### Analyses

Each of the five datasets was first tested for any over-represented MeSH terms and GO terms. MeSH ORA was performed using the MeSH ORA Framework which includes the “meshr” and “MeSH.Zma.e.g.db” R-packages [14], the latter of which is a mapping table that connects gene Entrez Gene IDs to MeSH IDs. These packages can be installed using Bioconductor by running the command, “source(“https://bioconductor.org/biocLite.R”)”, followed by “biocLite(“meshr”)” and “biocLite(“MeSH.Zma.e.g.db”)”. Further instructions to install and run these packages are provided in Additional files 1, 2, 3, 4 and 5. Unfortunately, the majority of maize genes annotated in the maize version 2 reference genome [25] do not have a corresponding Entrez Gene ID, and therefore are not useful for MeSH analyses. Of the 40,481 gene models available from Ensembl Plants [26], only 14,142 have corresponding Entrez IDs. The “meshHyperGTest” function was implemented to conduct a hypergeometric test. Specifically, to test the probability that a specific MeSH term is enriched in a particular set of genes, as compared to a background gene set, this function calculates

$$P\left( {enrichment} \right) = \mathop \sum \limits_{x = s}^{{{ \hbox{min} }\left( {M,k} \right)}} \frac{{\left( {\begin{array}{*{20}c} M \\ x \\ \end{array} } \right)\left( {\begin{array}{*{20}c} {N - M} \\ {k - x} \\ \end{array} } \right)}}{{\left( {\begin{array}{*{20}c} N \\ k \\ \end{array} } \right)}},$$

where N is the total number of background genes, k is the number of genes in the set being tested, M is the number of background genes corresponding to the particular MeSH term, and s is the number of genes in the test set that correspond to that MeSH term [14]. For this study, all Entrez genes in the maize reference genome version 2 [25] were used as the background gene set. GO ORA was conducted using a similar approach, as demonstrated in the additional files. The necessary GOstats package, which requires a list of Entrez Gene IDs as input, is installed by running “biocLite(“GOstats”)”.

Next, semantic similarity between distinct experiments was evaluated using the MeSHSim R package [21] to elucidate if there are underlying relationships between the trait data-sets (seed size, ear number, or inflorescence traits) and the process data-sets (domestication, improvement), as well as the relationships within the process and trait datasets. The “headingSetSim” function was used, and results were plotted with the corrplot R package [27].

## Results

### Overrepresentation analysis

MeSH ORA involves performing a hypergeometric test to determine which MeSH terms are enriched among the candidate set of genes compared to a set of background genes. All genes in the maize reference genome version 2 [25] with Entrez Gene IDs were used as the background set. While GO terms are classified into the three groups “molecular function”, “cellular components”, and “biological processes”, MeSH classifications include several groups, many of which are geared more toward indexing biomedical manuscripts than biological processes. However, classifications including “chemicals and drugs”, “diseases”, “anatomy”, and “phenomena and processes”, all have the potential to contribute to the biological understanding of sets of genes. Counts of the number of overrepresented terms in three classification groups for MeSH and GO are provided in Table 2. The precise overrepresented terms in each of these categories for the five analyzed datasets are described in Additional files 1, 2, 3, 4 and 5. For the purpose of demonstration, MeSH terms identified within the “anatomy” classification are provided as an example and described in detail in Table 3. Many of the enriched terms serve to provide additional evidence for reasonable a priori expectations, such as the observation that “flowers” and “seeds” are both enriched within the set of genes under selection during domestication. However, others introduce interesting questions that could serve to drive hypothesis generation for future studies. For instance, the only enriched term identified from the ear number dataset is “endosperm”, which one would not immediately assume to be related to ear number.

**Table 2:** Number of MeSH and GO terms identified within three classification groups for both MeSH and GO

	Domestication	Improvement	Seed size	Ear number	Inflorescence traits
*MeSH category*					
Chemicals and drugs	18	19	11	0	13
Anatomy	5	7	3	1	4
Phenomena and processes	30	8	18	1	11
*GO category*					
Biological processes	52	48	59	28	72
Molecular function	27	37	20	17	33
Cellular components	12	15	14	6	8

**Table 3:** MeSH terms enriched in each of the five datasets within the “anatomy” MeSH classification group

Domestication	Improvement	Seed size	Ear number	Inflorescence traits
*MeSH terms*				
Chromosomes Centromere Flowers Seeds Cyto. vesicles	Xylem Phloem Chromosomes golgi Apparatus Cyto. vesicles Ribosomes Flowers	Cytosol Shoots Chromosomes	Endosperm	Endo. reticulum Cell membrane Plant leaves Thylakoids

### Semantic similarity analysis

**Fig. 1:**
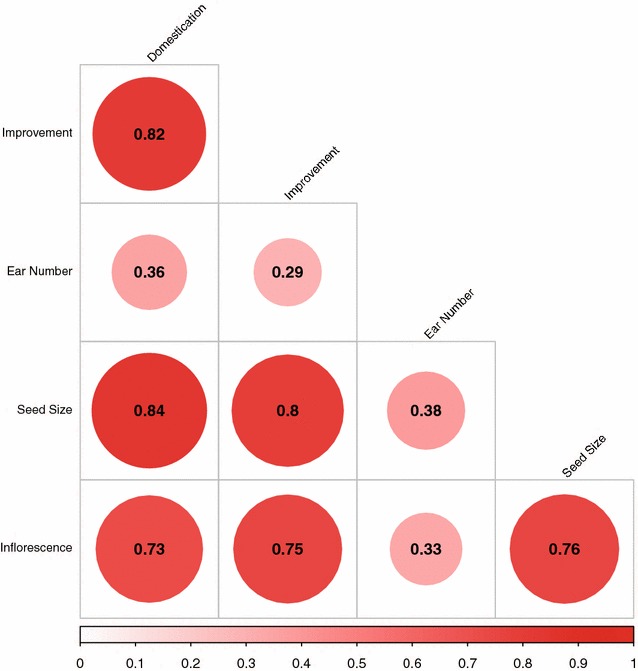
MeSH semantic similarity-based relatedness among sets of genes implicated in each of the five datasets studied. The size of each *circle*, degree of *red shading*, and value reported correspond to the relatedness between each pair of datasets

Another powerful use of MeSH is that it can be used to calculate the semantic similarity between distinct sets of MeSH terms. This type of analysis enables one to look for hidden relationships among sets of genes, potentially uncovering biological meaning. For the five datasets we studied, we assessed whether there were pairwise relationships linking any of them. Figure 1 depicts the MeSH similarity between each set of candidate genes. Interestingly, the strongest relationship identified was between domestication genes and seed size genes, possibly suggesting that seed size traits were more strongly selected during domestication than were ear number or other inflorescence traits. Noteworthy relationships were also observed between domestication and improvement genes, as well as between seed size and improvement genes. It should be noted that ear number genes were not strongly related to any of the other gene sets, which may simply result from the fact that the ear number dataset included the fewest candidate genes. This possibility is elaborated upon further in the discussion.

### Comparison of real data to a random set of genes

We conducted an analysis of 1500 randomly selected genes to determine the robustness of MeSH analyses in a scenario where no biological meaning is present (Additional file 6). As is expected for any *p* value based method, a subset of terms achieved significance. Spurious results were also observed in a parallel GO analysis (Additional file 6). In contrast to many of the real datasets we evaluated, there was no overwhelming theme tying the terms together. This subjective observation is supported by a semantic similarity analysis between the random gene set and the real datasets, where lower similarities were generally observed (Additional file 7). Still, the observation that “significant” MeSH or GO terms can arise from a random set of genes suggests that caution should be exercised when attempting to make interpretations from any such study, as is discussed in detail by Pavlidis et al. [28]. Although we utilized a lenient p = 0.05 significance threshold here, in part for the purpose of demonstration, the use of a hypergeometric distribution for testing allows a more stringent significance threshold to be employed when needed.

## Discussion

Our analysis of five existing datasets demonstrates how MeSH ORA and semantic-similarity analyses can be used to mine data and confirm and/or generate informative hypotheses. Like GO, MeSH-based approaches leverage curated annotations to provide biological insights. In fact, as we have shown, several of the enriched terms within the “anatomy” category are directly related to macro phenotypes, such as “seeds”, “shoots”, “flowers”, and “ears”. Whether applied to existing data, as we have demonstrated here, or if used to infer meaning from a list of candidates generated from a novel mapping study, MeSH represents an additional tool for drawing inferences from large-scale sets of genomic data.

### Biological implications

Among the findings gleaned from this analysis was the observation that while both “flowers” and “seeds” were enriched terms in the domestication set of genes, only “flowers” remained significant among improvement genes (Table 3). This result is consistent with the morphological observation that the maize female inflorescence is dramatically different from that of teosinte [29], with one of the most immediately apparent differences being seed related; the teosinte outer glume forms a hard teosinte fruitcase that completely encapsulates each kernel, while in maize the outer glume is barely present [30]. It has been shown that this trait is controlled by relatively few genes, with tga1 [31, 32] being of particular importance, and therefore our MeSH finding may suggest that after intense selection on seed traits during domestication, subsequent selection on further seed modifications during improvement has possibly been more subdued.

The hypothesis that domestication immediately impacted seed-related traits more than others is further supported by our semantic similarity analysis, where the most similar pair of gene-sets we tested corresponded to domestication and seed size (Fig. 1). Also, while the limited number of genes included in the ear-number dataset [19] seems to constrain the estimated similarity between ear-number genes and the other datasets, we do observe that ear-number genes are semantically more similar to domestication genes than they are to improvement genes (Fig. 1). This again is consistent with morphological differences between maize and teosinte, with maize demonstrating apical dominance while teosinte has a much more branched structure [33]. The observation of greater similarity between ear number genes and domestication genes than between ear number genes and improvement genes lends support to the existing supposition that single-eared plants have likely been favorable throughout the era of post-domestication maize improvement due to the ease with which such plants can be hand harvested [34].

An observation that ran contrary to our expectation was that “shoots” was an enriched term among seed size genes, while “endosperm” was enriched within the set of ear number genes (Table 3). We are tempted to dismiss these findings as spurious, but both have plausible biological explanations. In the Krug selection population [18], where our seed size regions were identified, mass selection not only impacted seed size, but also affected seedling size, leaf width, stalk circumference, and cob weight [35], indicating that the set of genes selected for seed size also being implicated in shoot traits is not unexpected. Similarly, the ear number genes were identified from the Golden Glow selection experiment for ear number [36], where correlated changes in kernel size and kernel number were also observed [34].

### Comparison of MeSH and GO overrepresentation analyses

Among the most obvious findings when comparing results from MeSH and GO for all five of the datasets is that the number of GO term associations dramatically surpasses the number identified by MeSH (Table 2). Within the sets of overrepresented terms (Additional files 1, 2, 3, 4, 5), there are cases of clearly overlapping GO and MeSH terms. For instance, in the improvement dataset, MeSH identified “Lipoxygenase” as the most significantly overrepresented term in the Chemicals and Drugs category, while GO identified the similar “linoleate 13S-lipoxygenase activity” term as highly significant in the Molecular Function category. However, there were instances where the MeSH analysis identified associations that were missed by GO. An example of this is that from the inflorescence dataset “Hybrid Vigor” was an enriched term in the Phenomena and Processes MeSH category, while no similar terms were identified by GO in any category. Although these examples are anecdotal, they are only a minor subset of the complete lists provided by this analysis and available for further scrutiny (Additional files 1, 2, 3, 4, 5). We mention the examples to demonstrate that MeSH and GO can either differ remarkably in their findings or, in some instances, particularly for highly significant terms, provide an independent confirmation that the other method is on the right track.

The most meaningful difference between MeSH and GO analyses is the source from which the annotations are derived. While most GO annotations are assigned algorithmically [37] with little or no human input [38], MeSH annotations are derived from manually curated manuscript classifications. This difference seems to lead to the existence of MeSH terms that correspond to easily interpretable macro-scale phenotypes, but it introduces additional complications as well. For example, the mention of a specific gene in a manuscript about hybrid-vigor may lead to a MeSH annotation of “hybrid-vigor” for that gene, even if no direct link was implied by the authors. However, this is a consideration that should always be at the forefront of ORA, regardless of the annotation scheme being used. To summarize, since MeSH and GO analyses are based on wholly different annotation mechanisms, the two approaches have the potential complement one another nicely. It is not our intention to suggest that MeSH should supplant GO, or even be viewed as a competitor to GO, since both platforms can provide distinct insights.

## Current limitations

Despite the promising MeSH ORA and semantic similarity results observed in this study, using MeSH to guide biological interpretations still has an assortment of limitations that should be considered during any study that involves MeSH. Firstly, for non-model organisms, including maize and other crops, relatively few genes have corresponding manuscripts that have been directly annotated with MeSH terms. Additionally, due to the nature of NCBI-based annotations, a requirement of current software is that all genes have Entrez Gene ID’s [39] to enable mapping from genes to MeSH terms, but Entrez Gene ID’s have only been assigned to a subset of maize genes. In fact, among the five datasets we analyzed, approximately two-thirds of the genes falling within the putatively functional regions did not have a corresponding Entrez Gene ID. This is particularly troubling in light of our observations regarding the ear number gene set, which was the smallest list of genes considered. Only 195 genes were contained within the selected regions (compared to thousands for some of the other data sets), and only 62 of those had corresponding Entrez Gene IDs. With fewer genes included during ORA, the power to detect significant enrichment is reduced. Similarly, this dataset showed very weak similarity to the others, which we hypothesize is at least in part due to the limited number of included genes and corresponding MeSH terms.

## Conclusions

Even considering the above limitations, we expect MeSH-based analyses will improve over time. As additional mapping and functional manuscripts are published, the number of Entrez genes and the descriptive MeSH terms corresponding to each, in both model and non-model species, will increase. This increase will improve the magnitude and reliability of results gleaned from MeSH. Although improvements are expected with time, the five datasets studied here demonstrate how MeSH can currently be leveraged for making biological interpretations in maize as well as other crop and plant species.

## Additional files

**Additional file 1** R-Markdown file including script and results of MeSH and GO analysis on maize domestication genes.

**Additional file 2** R-Markdown file including script and results of MeSH and GO analysis on maize improvement genes.

**Additional file 3** R-Markdown file including script and results of MeSH and GO analysis on maize genes under selection for an increase in maize seed size.

**Additional file 4** R-Markdown file including script and results of MeSH and GO analysis on maize genes under selection for an increase in ear number per plant.

**Additional file 5** R-Markdown file including script and results of MeSH and GO analysis on maize genes implicated in a GWAS study of maize inflorescence traits.

**Additional file 6** R-Markdown file including script and results of MeSH and GO analysis on a random set of 1500 maize genes.

**Additional file 7** R-Markdown file including script and results of MeSH semantic similarity analysis.

## Abbreviations

GWAS: genome wide association study
MeSH: Medical Subject Headings
NLM: National Library of Medicine
ORA: overrepresentation analysis
