# Supplementary material for: Medical Subject Heading (MeSH) annotations illuminate maize genetics and evolution
Source: Plant Methods. 2017 Feb 23;13:8. doi: 10.1186/s13007-017-0159-5 (PMC5324291; doi:10.1186/s13007-017-0159-5)
Supplement: Supplementary file 1 — Additional file 1 R-Markdown file including script and results of MeSH and GO analysis on maize domestication genes. [file 13007_2017_159_MOESM1_ESM.html]

MeSH over-representation analysis (Maize Domestication)


# MeSH over-representation analysis (Maize Domestication)

## 0. Install BiomaRt

```
#source("https://bioconductor.org/biocLite.R")
#biocLite("biomaRt")
setwd("/home/beissinger/Documents/MESH_Maize/Manuscript/Supplemental Data/")
```

## 1. Create a vector of background genes

We first create a vector of background genes. We will use every gene with the required data (entrez id) as the background.

```
library(biomaRt)
## access to biomaRt
mart <- useMart(biomart = "plants_mart", host="plants.ensembl.org", dataset="zmays_eg_gene")
univ.geneID <- getBM(attributes=c("ensembl_gene_id", "entrezgene"), mart = mart) # 40481
## remove genes with no corresponding Entrez Gene ID
univ.geneID2 <- univ.geneID[!is.na(univ.geneID[,2]),] # 14142 
## remove duplicated Entrez Gene ID
univ.geneID3 <- univ.geneID2[ !duplicated(univ.geneID2[,2]),] # 13630
##Get GO terms
univ.geneID4<-getBM(attributes=c("entrezgene","go_accession","go_name_1006","go_namespace_1003","go_linkage_type"),mart=mart,filters='entrezgene',values=univ.geneID3$entrezgene)
## Code evidence for genes without GO terms as NA
univ.geneID5<-univ.geneID4[-which(univ.geneID4[,2]==""),]
###Make dataframe for GOStats
goframeData <- data.frame(go_id = univ.geneID5$go_accession, Evidence = univ.geneID5$go_linkage_type, gene_id = univ.geneID5$entrezgene,stringsAsFactors=F)
```

# 2. Create a vector of selected genes

Secondly, we create a vector of significant genes by reading the input file: Domestication candidates from Hufford, 2012.

```
## read data
my.geneID <- read.csv("/home/beissinger/Documents/MESH_Maize/Hufford_Supp/Hufford_S2_Domestication.csv", header=T,stringsAsFactors = F)
my.geneID <- my.geneID[,1:5]
colnames(my.geneID)[1] <- "ensembl_gene_id"
## merge two files
my.geneID2 <- merge(my.geneID, univ.geneID3, by ="ensembl_gene_id") # 620
## remove duplicated Entrez Gene ID
my.geneID3 <- my.geneID2[ !duplicated(my.geneID2$entrezgene),] # 620
```

## 3. GO enrichment analysis

We can perform a GO analysis using the *GOstats* package. Code to perform it is below.

```
#source("https://bioconductor.org/biocLite.R")
#biocLite("GOstats")
#biocLite("GOSemSim")
#biocLite("AnnotationForge")
#library("AnnotationForge")
#available.dbschemas() #maize is not available :-(
library("GOstats")
library("GOSemSim")
##Prepare GO to gene mappings
goFrame=GOFrame(goframeData,organism="Zea mays")
goAllFrame=GOAllFrame(goFrame)
library(GSEABase)
gsc <- GeneSetCollection(goAllFrame, setType = GOCollection())

params <- GSEAGOHyperGParams(name="Domestication Zea mays GO", geneSetCollection=gsc, geneIds = my.geneID3[,6],
                             universeGeneIds = univ.geneID5$entrezgene, ontology = "BP", pvalueCutoff = 0.05, conditional = TRUE,
                             testDirection = "over")
```

GO enrichment analysis for **BP**.

```
BP <- hyperGTest(params)
summary(BP)[,c(1,2,7)] # 50
```

```
##        GOBPID      Pvalue
## 1  GO:0044723 0.001305638
## 2  GO:0016024 0.001541595
## 3  GO:0018342 0.001541595
## 4  GO:0044262 0.005348111
## 5  GO:0046855 0.005654002
## 6  GO:0080022 0.005863320
## 7  GO:0030244 0.006899941
## 8  GO:0009744 0.008589903
## 9  GO:0000956 0.010291180
## 10 GO:0042753 0.010978486
## 11 GO:0048574 0.010978486
## 12 GO:0043647 0.014498072
## 13 GO:0040029 0.014512804
## 14 GO:0072529 0.017766260
## 15 GO:0010152 0.017766260
## 16 GO:0009901 0.017766260
## 17 GO:0048573 0.021703520
## 18 GO:0009743 0.022597705
## 19 GO:0090305 0.024636481
## 20 GO:0046031 0.025540953
## 21 GO:0009185 0.025540953
## 22 GO:0009135 0.025540953
## 23 GO:0006096 0.025540953
## 24 GO:0048443 0.029284054
## 25 GO:0009749 0.029284054
## 26 GO:0046351 0.031096498
## 27 GO:0006165 0.033907713
## 28 GO:0051345 0.035186085
## 29 GO:0046174 0.035186085
## 30 GO:0005978 0.035186085
## 31 GO:0048511 0.037010276
## 32 GO:0009900 0.043888815
## 33 GO:0010325 0.044125465
## 34 GO:0010199 0.044125465
## 35 GO:0010165 0.044125465
## 36 GO:0042407 0.044125465
## 37 GO:0051776 0.044125465
## 38 GO:0019401 0.044125465
## 39 GO:0032467 0.044125465
## 40 GO:0000719 0.044125465
## 41 GO:0019593 0.044125465
## 42 GO:0018023 0.044125465
## 43 GO:0048859 0.044125465
## 44 GO:0046294 0.044125465
## 45 GO:0006059 0.044125465
## 46 GO:2000694 0.044125465
## 47 GO:0018106 0.044125465
## 48 GO:0009094 0.045568123
## 49 GO:1901616 0.045568123
## 50 GO:0006112 0.045568123
## 51 GO:0051273 0.046806857
## 52 GO:0034655 0.046806857
##                                                                    Term
## 1                        single-organism carbohydrate metabolic process
## 2                               CDP-diacylglycerol biosynthetic process
## 3                                                   protein prenylation
## 4                               cellular carbohydrate metabolic process
## 5                                  inositol phosphate dephosphorylation
## 6                                              primary root development
## 7                                        cellulose biosynthetic process
## 8                                                   response to sucrose
## 9                            nuclear-transcribed mRNA catabolic process
## 10                              positive regulation of circadian rhythm
## 11                                   long-day photoperiodism, flowering
## 12                                 inositol phosphate metabolic process
## 13                            regulation of gene expression, epigenetic
## 14                     pyrimidine-containing compound catabolic process
## 15                                                    pollen maturation
## 16                                                    anther dehiscence
## 17                                            photoperiodism, flowering
## 18                                             response to carbohydrate
## 19                          nucleic acid phosphodiester bond hydrolysis
## 20                                                ADP metabolic process
## 21                         ribonucleoside diphosphate metabolic process
## 22                      purine nucleoside diphosphate metabolic process
## 23                                                   glycolytic process
## 24                                                   stamen development
## 25                                                  response to glucose
## 26                                    disaccharide biosynthetic process
## 27                               nucleoside diphosphate phosphorylation
## 28                            positive regulation of hydrolase activity
## 29                                             polyol catabolic process
## 30                                        glycogen biosynthetic process
## 31                                                     rhythmic process
## 32                                                           dehiscence
## 33                raffinose family oligosaccharide biosynthetic process
## 34 organ boundary specification between lateral organs and the meristem
## 35                                                    response to X-ray
## 36                                                    cristae formation
## 37                                             detection of redox state
## 38                                         alditol biosynthetic process
## 39                                   positive regulation of cytokinesis
## 40                                                 photoreactive repair
## 41                                        mannitol biosynthetic process
## 42                                       peptidyl-lysine trimethylation
## 43                                     formation of anatomical boundary
## 44                                       formaldehyde catabolic process
## 45                                            hexitol metabolic process
## 46                  regulation of phragmoplast microtubule organization
## 47                                   peptidyl-histidine phosphorylation
## 48                                 L-phenylalanine biosynthetic process
## 49                           organic hydroxy compound catabolic process
## 50                                     energy reserve metabolic process
## 51                                        beta-glucan metabolic process
## 52                     nucleobase-containing compound catabolic process
```

```
# GO similarity
#install.packages("corrplot")
library(corrplot)
#goListBP <- summary(BP)[,c(1)]
#goSimMatBP <- mgoSim(goListBP, goListBP, ont="BP", measure="Wang", combine=NULL)
#corrplot(goSimMatBP, is.corr = FALSE, type="lower", tl.col = "black", tl.cex = 0.8)
```

GO enrichment analysis for **MF**

```
ontology(params) <- "MF"
MF <- hyperGTest(params)
summary(MF)[,c(1,2,7)] # 26
```

```
##        GOMFID       Pvalue
## 1  GO:0047274 0.0003999399
## 2  GO:0052866 0.0003999399
## 3  GO:0008318 0.0018623972
## 4  GO:0004805 0.0124527513
## 5  GO:0016779 0.0171336532
## 6  GO:0019200 0.0180418532
## 7  GO:0004605 0.0201113370
## 8  GO:0047334 0.0201113370
## 9  GO:0008878 0.0201113370
## 10 GO:0004664 0.0292358070
## 11 GO:0051087 0.0308978035
## 12 GO:0060590 0.0396718416
## 13 GO:0008199 0.0470775529
## 14 GO:0015633 0.0470775529
## 15 GO:0004322 0.0470775529
## 16 GO:0018738 0.0470775529
## 17 GO:0004474 0.0470775529
## 18 GO:0004164 0.0470775529
## 19 GO:0015434 0.0470775529
## 20 GO:0080039 0.0470775529
## 21 GO:0003904 0.0470775529
## 22 GO:0008531 0.0470775529
## 23 GO:0018392 0.0470775529
## 24 GO:0042389 0.0470775529
## 25 GO:0031386 0.0470775529
## 26 GO:0003837 0.0470775529
## 27 GO:0030234 0.0486879681
##                                                              Term
## 1               galactinol-sucrose galactosyltransferase activity
## 2             phosphatidylinositol phosphate phosphatase activity
## 3                              protein prenyltransferase activity
## 4                                  trehalose-phosphatase activity
## 5                                 nucleotidyltransferase activity
## 6                                    carbohydrate kinase activity
## 7                     phosphatidate cytidylyltransferase activity
## 8  diphosphate-fructose-6-phosphate 1-phosphotransferase activity
## 9                glucose-1-phosphate adenylyltransferase activity
## 10                                prephenate dehydratase activity
## 11                                              chaperone binding
## 12                                      ATPase regulator activity
## 13                                            ferric iron binding
## 14                              zinc transporting ATPase activity
## 15                                           ferroxidase activity
## 16                         S-formylglutathione hydrolase activity
## 17                                       malate synthase activity
## 18                                    diphthine synthase activity
## 19                           cadmium-transporting ATPase activity
## 20                       xyloglucan endotransglucosylase activity
## 21                     deoxyribodipyrimidine photo-lyase activity
## 22                                     riboflavin kinase activity
## 23             glycoprotein 3-alpha-L-fucosyltransferase activity
## 24                         omega-3 fatty acid desaturase activity
## 25                                                    protein tag
## 26                                 beta-ureidopropionase activity
## 27                                      enzyme regulator activity
```

```
# GO similarity
#goListMF <- summary(MF)[,c(1)]
#goSimMatMF <- mgoSim(goListMF, goListMF, ont="MF", measure="Wang", combine=NULL)
#corrplot(goSimMatMF, is.corr = FALSE, type="lower", tl.col = "black", tl.cex = 0.8,)
```

GO enrichment analysis for **CC**

```
ontology(params) <- "CC"
CC <- hyperGTest(params)
summary(CC)[,c(1,2,7)] # 12
```

```
##        GOCCID      Pvalue
## 1  GO:0005762 0.005040924
## 2  GO:0005759 0.005734820
## 3  GO:0005956 0.015894409
## 4  GO:0043233 0.024734227
## 5  GO:0061695 0.030212242
## 6  GO:0030915 0.031585643
## 7  GO:0000812 0.041646332
## 8  GO:0032578 0.041646332
## 9  GO:0005828 0.041646332
## 10 GO:0016363 0.041646332
## 11 GO:0016442 0.041646332
## 12 GO:0043234 0.046019274
##                                                              Term
## 1                           mitochondrial large ribosomal subunit
## 2                                            mitochondrial matrix
## 3                                      protein kinase CK2 complex
## 4                                                 organelle lumen
## 5  transferase complex, transferring phosphorus-containing groups
## 6                                               Smc5-Smc6 complex
## 7                                                    Swr1 complex
## 8                                         aleurone grain membrane
## 9                                         kinetochore microtubule
## 10                                                 nuclear matrix
## 11                                                   RISC complex
## 12                                                protein complex
```

```
# GO similarity
#goListCC <- summary(CC)[,c(1)]
#goSimMatCC <- mgoSim(goListCC, goListCC, ont="CC", measure="Wang", combine=NULL,organism="maize")
#corrplot(goSimMatCC, is.corr = FALSE, type="lower", tl.col = "black", tl.cex = 0.8)
```

## 4. MeSH enrichment analysis

Then, we perform a MeSH ORA for the category **Chemicals and Drugs** by setting ‘category=“D”’. Different categories are set as different letters, as will become clear in the following sections.

```
source("https://bioconductor.org/biocLite.R")
#biocLite("meshr")
#biocLite("MeSH.db")
#biocLite("MeSH.Zma.eg.db")
#biocLite("MeSHSim")
library(meshr)
library(MeSH.db)
library("MeSH.Zma.eg.db")
meshParams <- new("MeSHHyperGParams", geneIds = my.geneID3[,6], universeGeneIds = univ.geneID3[,2], 
                  annotation = "MeSH.Zma.eg.db", category = "D", database = "gene2pubmed", 
                  pvalueCutoff = 0.05, pAdjust = "none")
meshR <- meshHyperGTest(meshParams)
summary(meshR)[!duplicated(summary(meshR)[,7]),c(1,2,7)]
```

```
##         MESHID       Pvalue                                     MESHTERM
## 6      D004251 0.0004705092                    DNA Transposable Elements
## 90060  D018076 0.0020607780                           DNA, Complementary
## 1      D000226 0.0020659628          Mitochondrial ADP, ATP Translocases
## 154011 D035683 0.0069713393                                    MicroRNAs
## 119745 D018626 0.0070456659                                Retroelements
## 142568 D018749 0.0071540082                                   RNA, Plant
## 131038 D018744 0.0089103658                                   DNA, Plant
## 165658 D051599 0.0116590073      Glucose-1-Phosphate Adenylyltransferase
## 44697  D011061 0.0177853289                                       Poly A
## 27130  D010743 0.0188487517                                Phospholipids
## 60487  D014157 0.0235344655                        Transcription Factors
## 76518  D017931 0.0384314684                                  DNA Primers
## 27140  D010940 0.0429618625                               Plant Proteins
## 27128  D006836 0.0454878944                                 Hydro-Lyases
## 27129  D008081 0.0454878944                                    Liposomes
## 60484  D012967 0.0454878944                       Sodium Dodecyl Sulfate
## 154007 D026342 0.0454878944                 AGAMOUS Protein, Arabidopsis
## 165657 D051103 0.0454878944 1-Acylglycerol-3-Phosphate O-Acyltransferase
```

```
# Store list of terms
headingListD <- summary(meshR)[!duplicated(summary(meshR)[,7]),c(7)]
```

Switching to a different category is easily done by the ‘category<-’ function. Here, we use **Diseases** (category = “C”).

```
category(meshParams) <- "C"
meshR <- meshHyperGTest(meshParams)
summary(meshR)[!duplicated(summary(meshR)[,7]),c(1,2,7)]
```

```
##    MESHID     Pvalue   MESHTERM
## 1 D011123 0.04548789 Polyploidy
```

```
# Store list of terms
headingListC <- summary(meshR)[!duplicated(summary(meshR)[,7]),c(7)]
```

MeSH ORA for **Anatomy** (category = “A”).

```
category(meshParams) <- "A"
meshR <- meshHyperGTest(meshParams)
summary(meshR)[!duplicated(summary(meshR)[,7]),c(1,2,7)]
```

```
##        MESHID       Pvalue             MESHTERM
## 11559 D032461 0.0004616885   Chromosomes, Plant
## 38705 D035264 0.0052472546              Flowers
## 1     D002503 0.0068977091           Centromere
## 11286 D012639 0.0257363443                Seeds
## 11558 D022162 0.0454878944 Cytoplasmic Vesicles
```

```
# Store list of terms
headingListA <- summary(meshR)[!duplicated(summary(meshR)[,7]),c(7)]
```

MeSH ORA for **Phenomena and Processes** (category = “G”).

```
category(meshParams) <- "G"
meshR <- meshHyperGTest(meshParams)
summary(meshR)[!duplicated(summary(meshR)[,7]),c(1,2,7)]
```

```
##         MESHID       Pvalue                        MESHTERM
## 321696 D032461 0.0004616885              Chromosomes, Plant
## 54987  D004251 0.0004705092       DNA Transposable Elements
## 275248 D020224 0.0013770421         Expressed Sequence Tags
## 1      D001483 0.0030370439                   Base Sequence
## 213740 D017434 0.0045438091     Protein Structure, Tertiary
## 43702  D002503 0.0068977091                      Centromere
## 93457  D011003 0.0068977091                        Ploidies
## 348842 D056915 0.0068977091      DNA Copy Number Variations
## 82110  D007178 0.0069713393                      Inbreeding
## 240881 D018626 0.0070456659                   Retroelements
## 213813 D018556 0.0071206945             Crops, Agricultural
## 263938 D019175 0.0071206945                 DNA Methylation
## 118601 D011995 0.0074279396          Recombination, Genetic
## 146855 D014644 0.0075859199               Genetic Variation
## 158398 D017343 0.0076155471                    Genes, Plant
## 252174 D018745 0.0109163418                   Genome, Plant
## 305159 D020641 0.0112497046 Polymorphism, Single Nucleotide
## 321514 D026801 0.0114802052                         Synteny
## 225109 D018598 0.0168319011           Minisatellite Repeats
## 321493 D023061 0.0188487517                      Gene Order
## 129907 D012639 0.0257363443                           Seeds
## 130179 D012689 0.0306628356 Sequence Homology, Nucleic Acid
## 104739 D011401 0.0361928775       Promoter Regions, Genetic
## 200259 D017398 0.0377590492            Alternative Splicing
## 82109  D005720 0.0454878944                      Gamma Rays
## 104737 D011123 0.0454878944                      Polyploidy
## 213812 D018257 0.0454878944         Helix-Loop-Helix Motifs
## 360122 D057895 0.0454878944              Haploinsufficiency
## 93404  D010766 0.0454951398                 Phosphorylation
## 275233 D019521 0.0482030685                 Body Patterning
```

```
# Store list of terms
headingListG <- summary(meshR)[!duplicated(summary(meshR)[,7]),c(7)]
```

## 5. Output list of significant MeSH headers

```
DomesticationMeshList <- list(headingListA,headingListC,headingListD,headingListG)
save(DomesticationMeshList,file="DomesticationMeshList.Robj")
```

## 6. Session Information

```
sessionInfo()
```

```
## R version 3.3.0 (2016-05-03)
## Platform: x86_64-redhat-linux-gnu (64-bit)
## Running under: Fedora 23 (Workstation Edition)
## 
## locale:
##  [1] LC_CTYPE=en_US.UTF-8       LC_NUMERIC=C              
##  [3] LC_TIME=en_US.UTF-8        LC_COLLATE=en_US.UTF-8    
##  [5] LC_MONETARY=en_US.UTF-8    LC_MESSAGES=en_US.UTF-8   
##  [7] LC_PAPER=en_US.UTF-8       LC_NAME=C                 
##  [9] LC_ADDRESS=C               LC_TELEPHONE=C            
## [11] LC_MEASUREMENT=en_US.UTF-8 LC_IDENTIFICATION=C       
## 
## attached base packages:
##  [1] grid      stats4    parallel  stats     graphics  grDevices utils    
##  [8] datasets  methods   base     
## 
## other attached packages:
##  [1] MeSH.Zma.eg.db_1.6.0     meshr_1.8.0             
##  [3] MeSH.Syn.eg.db_1.6.0     MeSH.Bsu.168.eg.db_1.6.0
##  [5] MeSH.Aca.eg.db_1.6.0     MeSH.Hsa.eg.db_1.6.0    
##  [7] MeSH.PCR.db_1.6.0        MeSH.AOR.db_1.6.0       
##  [9] MeSH.db_1.6.0            MeSHDbi_1.8.0           
## [11] org.Hs.eg.db_3.3.0       cummeRbund_2.14.0       
## [13] Gviz_1.16.1              rtracklayer_1.32.1      
## [15] GenomicRanges_1.24.2     GenomeInfoDb_1.8.1      
## [17] fastcluster_1.1.20       reshape2_1.4.1          
## [19] ggplot2_2.1.0            fdrtool_1.2.15          
## [21] BiocInstaller_1.22.3     corrplot_0.77           
## [23] GSEABase_1.34.0          annotate_1.50.0         
## [25] XML_3.98-1.4             GO.db_3.3.0             
## [27] RSQLite_1.0.0            DBI_0.4-1               
## [29] GOSemSim_1.30.2          GOstats_2.38.1          
## [31] graph_1.50.0             Category_2.38.0         
## [33] Matrix_1.2-6             AnnotationDbi_1.34.4    
## [35] IRanges_2.6.1            S4Vectors_0.10.2        
## [37] Biobase_2.32.0           BiocGenerics_0.18.0     
## [39] biomaRt_2.28.0          
## 
## loaded via a namespace (and not attached):
##  [1] bitops_1.0-6                  matrixStats_0.50.2           
##  [3] RColorBrewer_1.1-2            httr_1.2.1                   
##  [5] tools_3.3.0                   R6_2.1.2                     
##  [7] rpart_4.1-10                  Hmisc_3.17-4                 
##  [9] colorspace_1.2-6              nnet_7.3-12                  
## [11] gridExtra_2.2.1               chron_2.3-47                 
## [13] formatR_1.4                   scales_0.4.0                 
## [15] genefilter_1.54.2             RBGL_1.48.1                  
## [17] stringr_1.0.0                 digest_0.6.9                 
## [19] Rsamtools_1.24.0              foreign_0.8-66               
## [21] rmarkdown_1.0                 AnnotationForge_1.14.2       
## [23] XVector_0.12.0                dichromat_2.0-0              
## [25] htmltools_0.3.5               ensembldb_1.4.7              
## [27] BSgenome_1.40.1               shiny_0.13.2                 
## [29] BiocParallel_1.6.2            acepack_1.3-3.3              
## [31] VariantAnnotation_1.18.3      RCurl_1.95-4.8               
## [33] magrittr_1.5                  Formula_1.2-1                
## [35] Rcpp_0.12.5                   munsell_0.4.3                
## [37] stringi_1.1.1                 yaml_2.1.13                  
## [39] SummarizedExperiment_1.2.3    zlibbioc_1.18.0              
## [41] plyr_1.8.4                    AnnotationHub_2.4.2          
## [43] lattice_0.20-33               Biostrings_2.40.2            
## [45] splines_3.3.0                 GenomicFeatures_1.24.4       
## [47] knitr_1.13                    evaluate_0.9                 
## [49] biovizBase_1.20.0             latticeExtra_0.6-28          
## [51] data.table_1.9.6              httpuv_1.3.3                 
## [53] gtable_0.2.0                  mime_0.5                     
## [55] xtable_1.8-2                  survival_2.39-5              
## [57] GenomicAlignments_1.8.4       cluster_2.0.4                
## [59] interactiveDisplayBase_1.10.3
```
