# Supplementary material for: Medical Subject Heading (MeSH) annotations illuminate maize genetics and evolution
Source: Plant Methods. 2017 Feb 23;13:8. doi: 10.1186/s13007-017-0159-5 (PMC5324291; doi:10.1186/s13007-017-0159-5)
Supplement: Supplementary file 2 — Additional file 2 R-Markdown file including script and results of MeSH and GO analysis on maize improvement genes. [file 13007_2017_159_MOESM2_ESM.html]

MeSH over-representation analysis (Maize Improvement)


# MeSH over-representation analysis (Maize Improvement)

## 0. Install BiomaRt

```
#source("https://bioconductor.org/biocLite.R")
#biocLite("biomaRt")
setwd("/home/beissinger/Documents/MESH_Maize/Manuscript/Supplemental Data/")
```

## 1. Create a vector of background genes

We first create a vector of background genes. We will use every gene with the required data (entrez id) as the background.

```
library(biomaRt)
## access to biomaRt
mart <- useMart(biomart = "plants_mart", host="plants.ensembl.org", dataset="zmays_eg_gene")
univ.geneID <- getBM(attributes=c("ensembl_gene_id", "entrezgene"), mart = mart) # 40481
## remove genes with no corresponding Entrez Gene ID
univ.geneID2 <- univ.geneID[!is.na(univ.geneID[,2]),] # 14142 
## remove duplicated Entrez Gene ID
univ.geneID3 <- univ.geneID2[ !duplicated(univ.geneID2[,2]),] # 13630
##Get GO terms
univ.geneID4<-getBM(attributes=c("entrezgene","go_accession","go_name_1006","go_namespace_1003","go_linkage_type"),mart=mart,filters='entrezgene',values=univ.geneID3$entrezgene)
## Code evidence for genes without GO terms as NA
univ.geneID5<-univ.geneID4[-which(univ.geneID4[,2]==""),]
###Make dataframe for GOStats
goframeData <- data.frame(go_id = univ.geneID5$go_accession, Evidence = univ.geneID5$go_linkage_type, gene_id = univ.geneID5$entrezgene,stringsAsFactors=F)
```

# 2. Create a vector of selected genes

Secondly, we create a vector of significant genes by reading the input file: Improvement candidates from Hufford, 2012.

```
## read data
my.geneID <- read.csv("/home/beissinger/Documents/MESH_Maize/Hufford_Supp/Hufford_S3_Improvement.csv", header=T,stringsAsFactors = F)
my.geneID <- my.geneID[,1:5]
colnames(my.geneID)[1] <- "ensembl_gene_id"
## merge two files
my.geneID2 <- merge(my.geneID, univ.geneID3, by ="ensembl_gene_id") # 500
## remove duplicated Entrez Gene ID
my.geneID3 <- my.geneID2[ !duplicated(my.geneID2$entrezgene),] # 500
```

## 3. GO enrichment analysis

We can perform a GO analysis using the *GOstats* package. Code to perform it is below.

```
#source("https://bioconductor.org/biocLite.R")
#biocLite("GOstats")
#biocLite("GOSemSim")
#biocLite("AnnotationForge")
#library("AnnotationForge")
#available.dbschemas() #maize is not available :-(
library("GOstats")
library("GOSemSim")
##Prepare GO to gene mappings
goFrame=GOFrame(goframeData,organism="Zea mays")
goAllFrame=GOAllFrame(goFrame)
library(GSEABase)
gsc <- GeneSetCollection(goAllFrame, setType = GOCollection())

params <- GSEAGOHyperGParams(name="Domestication Zea mays GO", geneSetCollection=gsc, geneIds = my.geneID3[,6],
                             universeGeneIds = univ.geneID5$entrezgene, ontology = "BP", pvalueCutoff = 0.05, conditional = TRUE,
                             testDirection = "over")
```

GO enrichment analysis for **BP**

```
BP <- hyperGTest(params)
summary(BP)[,c(1,2,7)] # 42
```

```
##        GOBPID      Pvalue
## 1  GO:0048830 0.004240514
## 2  GO:0009753 0.006352414
## 3  GO:0042753 0.008267476
## 4  GO:0009860 0.008749852
## 5  GO:0031408 0.009351912
## 6  GO:0009856 0.010902982
## 7  GO:0006108 0.011819038
## 8  GO:0044403 0.013950882
## 9  GO:0046777 0.015973985
## 10 GO:0006812 0.018166578
## 11 GO:0045926 0.019645754
## 12 GO:0018342 0.019645754
## 13 GO:0010072 0.019645754
## 14 GO:0048511 0.023217943
## 15 GO:0046786 0.026818126
## 16 GO:0019058 0.026818126
## 17 GO:0009312 0.033845140
## 18 GO:0035821 0.033845140
## 19 GO:0010582 0.034868638
## 20 GO:0010020 0.034868638
## 21 GO:0006414 0.035549860
## 22 GO:0010381 0.038144604
## 23 GO:0044115 0.038144604
## 24 GO:0019427 0.038144604
## 25 GO:0019419 0.038144604
## 26 GO:0007631 0.038144604
## 27 GO:0019433 0.038144604
## 28 GO:0006516 0.038144604
## 29 GO:0051776 0.038144604
## 30 GO:0052805 0.038144604
## 31 GO:0046461 0.038144604
## 32 GO:0019556 0.038144604
## 33 GO:1903415 0.038144604
## 34 GO:0018315 0.038144604
## 35 GO:0052095 0.038144604
## 36 GO:0052096 0.038144604
## 37 GO:0031640 0.038144604
## 38 GO:0046503 0.038144604
## 39 GO:0044090 0.038144604
## 40 GO:0018106 0.038144604
## 41 GO:0044002 0.038144604
## 42 GO:0010028 0.038144604
## 43 GO:0006488 0.038144604
## 44 GO:0009100 0.041193232
## 45 GO:0006486 0.041763427
## 46 GO:0006542 0.043720344
## 47 GO:0009718 0.043720344
## 48 GO:0006465 0.043720344
##                                                                                                                 Term
## 1                                                                                      adventitious root development
## 2                                                                                          response to jasmonic acid
## 3                                                                            positive regulation of circadian rhythm
## 4                                                                                                 pollen tube growth
## 5                                                                                      oxylipin biosynthetic process
## 6                                                                                                        pollination
## 7                                                                                           malate metabolic process
## 8                                                               symbiosis, encompassing mutualism through parasitism
## 9                                                                                        protein autophosphorylation
## 10                                                                                                  cation transport
## 11                                                                                     negative regulation of growth
## 12                                                                                               protein prenylation
## 13                                                                       primary shoot apical meristem specification
## 14                                                                                                  rhythmic process
## 15                                                               viral replication complex formation and maintenance
## 16                                                                                                  viral life cycle
## 17                                                                              oligosaccharide biosynthetic process
## 18                                                        modification of morphology or physiology of other organism
## 19                                                                                       floral meristem determinacy
## 20                                                                                               chloroplast fission
## 21                                                                                          translational elongation
## 22                                                                           attachment of peroxisome to chloroplast
## 23                                                         development of symbiont involved in interaction with host
## 24                                                                      acetyl-CoA biosynthetic process from acetate
## 25                                                                                                 sulfate reduction
## 26                                                                                                  feeding behavior
## 27                                                                                    triglyceride catabolic process
## 28                                                                                    glycoprotein catabolic process
## 29                                                                                          detection of redox state
## 30                                                                   imidazole-containing compound catabolic process
## 31                                                                                   neutral lipid catabolic process
## 32                                                            histidine catabolic process to glutamate and formamide
## 33                                              flavonoid transport from endoplasmic reticulum to plant-type vacuole
## 34                                                    molybdenum incorporation into molybdenum-molybdopterin complex
## 35 formation of specialized structure for nutrient acquisition from other organism involved in symbiotic interaction
## 36                        formation by symbiont of syncytium involving giant cell for nutrient acquisition from host
## 37                                                                                killing of cells of other organism
## 38                                                                                    glycerolipid catabolic process
## 39                                                                       positive regulation of vacuole organization
## 40                                                                                peptidyl-histidine phosphorylation
## 41                                                                                acquisition of nutrients from host
## 42                                                                                                 xanthophyll cycle
## 43                                                              dolichol-linked oligosaccharide biosynthetic process
## 44                                                                                    glycoprotein metabolic process
## 45                                                                                             protein glycosylation
## 46                                                                                    glutamine biosynthetic process
## 47                                                              anthocyanin-containing compound biosynthetic process
## 48                                                                                         signal peptide processing
```

```
# GO similarity
library(corrplot)
#goListBP <- summary(BP)[,c(1)]
#goSimMatBP <- mgoSim(goListBP, goListBP, ont="BP", measure="Wang", combine=NULL)
#corrplot(goSimMatBP, is.corr = FALSE, type="lower", tl.col = "black", tl.cex = 0.8)
```

GO enrichment analysis for **MF**

```
ontology(params) <- "MF"
MF <- hyperGTest(params)
summary(MF)[,c(1,2,7)] # 37
```

```
##        GOMFID       Pvalue
## 1  GO:0004471 0.0004646556
## 2  GO:0004386 0.0014681736
## 3  GO:0008026 0.0014994090
## 4  GO:0016887 0.0019838868
## 5  GO:0015370 0.0039369008
## 6  GO:0008508 0.0039369008
## 7  GO:0042803 0.0056999004
## 8  GO:0008028 0.0076827324
## 9  GO:0047274 0.0076827324
## 10 GO:0016615 0.0106733140
## 11 GO:0016165 0.0124947836
## 12 GO:0005319 0.0160832089
## 13 GO:0050897 0.0180982127
## 14 GO:0008318 0.0182901599
## 15 GO:0016462 0.0223921871
## 16 GO:0051213 0.0271951954
## 17 GO:0016817 0.0309195589
## 18 GO:0015662 0.0325221783
## 19 GO:0004604 0.0367295884
## 20 GO:0004352 0.0367295884
## 21 GO:0009973 0.0367295884
## 22 GO:0016208 0.0367295884
## 23 GO:0008568 0.0367295884
## 24 GO:0030366 0.0367295884
## 25 GO:0004746 0.0367295884
## 26 GO:0018392 0.0367295884
## 27 GO:0046422 0.0367295884
## 28 GO:0046428 0.0367295884
## 29 GO:0030946 0.0367295884
## 30 GO:0004583 0.0367295884
## 31 GO:0008455 0.0367295884
## 32 GO:0050521 0.0367295884
## 33 GO:0008134 0.0408152331
## 34 GO:0004356 0.0408152331
## 35 GO:0016880 0.0408152331
## 36 GO:0042626 0.0441776429
## 37 GO:0016758 0.0459271653
##                                                                                     Term
## 1                                 malate dehydrogenase (decarboxylating) (NAD+) activity
## 2                                                                      helicase activity
## 3                                                        ATP-dependent helicase activity
## 4                                                                        ATPase activity
## 5                                                       solute:sodium symporter activity
## 6                                                    bile acid:sodium symporter activity
## 7                                                      protein homodimerization activity
## 8                                 monocarboxylic acid transmembrane transporter activity
## 9                                      galactinol-sucrose galactosyltransferase activity
## 10                                                         malate dehydrogenase activity
## 11                                                   linoleate 13S-lipoxygenase activity
## 12                                                            lipid transporter activity
## 13                                                                    cobalt ion binding
## 14                                                    protein prenyltransferase activity
## 15                                                              pyrophosphatase activity
## 16                                                                  dioxygenase activity
## 17                                         hydrolase activity, acting on acid anhydrides
## 18 ATPase activity, coupled to transmembrane movement of ions, phosphorylative mechanism
## 19                              phosphoadenylyl-sulfate reductase (thioredoxin) activity
## 20                                               glutamate dehydrogenase (NAD+) activity
## 21                                                   adenylyl-sulfate reductase activity
## 22                                                                           AMP binding
## 23                                                  microtubule-severing ATPase activity
## 24                                                       molybdopterin synthase activity
## 25                                                          riboflavin synthase activity
## 26                                    glycoprotein 3-alpha-L-fucosyltransferase activity
## 27                                                    violaxanthin de-epoxidase activity
## 28                             1,4-dihydroxy-2-naphthoate octaprenyltransferase activity
## 29                                protein tyrosine phosphatase activity, metal-dependent
## 30              dolichyl-phosphate-glucose-glycolipid alpha-glucosyltransferase activity
## 31        alpha-1,6-mannosylglycoprotein 2-beta-N-acetylglucosaminyltransferase activity
## 32                                                 alpha-glucan, water dikinase activity
## 33                                                          transcription factor binding
## 34                                                     glutamate-ammonia ligase activity
## 35                                               acid-ammonia (or amide) ligase activity
## 36                      ATPase activity, coupled to transmembrane movement of substances
## 37                                     transferase activity, transferring hexosyl groups
```

```
# GO similarity
#goListMF <- summary(MF)[,c(1)]
#goSimMatMF <- mgoSim(goListMF, goListMF, ont="MF", measure="Wang", combine=NULL)
#corrplot(goSimMatMF, is.corr = FALSE, type="lower", tl.col = "black", tl.cex = 0.8)
```

GO enrichment analysis for **CC**

```
ontology(params) <- "CC"
CC <- hyperGTest(params)
summary(CC)[,c(1,2,7)] # 15

# GO similarity
#goListCC <- summary(CC)[,c(1)]
#goSimMatCC <- mgoSim(goListCC, goListCC, ont="CC", measure="Wang", combine=NULL)
#corrplot(goSimMatCC[-4,-4], is.corr = FALSE, type="lower", tl.col = "black", tl.cex = 0.8)
```

## 4. MeSH enrichment analysis

Then, we perform a MeSH ORA for the category **Chemicals and Drugs** by setting ‘category=“D”’. Different categories are set as different letters, as will become clear in the following sections.

```
#biocLite("meshr")
#biocLite("MeSH.db")
#biocLite("MeSH.Zma.eg.db")
#biocLite("MeSHSim")
library(meshr)
library(MeSH.db)
library("MeSH.Zma.eg.db")
meshParams <- new("MeSHHyperGParams", geneIds = my.geneID3[,6], universeGeneIds = univ.geneID3[,2], 
                  annotation = "MeSH.Zma.eg.db", category = "D", database = "gene2pubmed", 
                  pvalueCutoff = 0.05, pAdjust = "none")
meshR <- meshHyperGTest(meshParams)
summary(meshR)[!duplicated(summary(meshR)[,7]),c(1,2,7)]
```

```
##        MESHID      Pvalue                                 MESHTERM
## 27131 D008084 0.007671371                             Lipoxygenase
## 27146 D014867 0.008428219                                    Water
## 40713 D020543 0.024952190                                 Proteome
## 1     D000348 0.036683786                               Aflatoxins
## 2     D000935 0.036683786                        Antifungal Agents
## 27127 D005634 0.036683786           Fructose-Bisphosphate Aldolase
## 27128 D008041 0.036683786                           Linoleic Acids
## 27129 D008054 0.036683786                          Lipid Peroxides
## 27130 D008081 0.036683786                                Liposomes
## 27143 D012967 0.036683786                   Sodium Dodecyl Sulfate
## 27165 D015800 0.036683786                       Protozoan Proteins
## 27168 D016337 0.036683786                           RNA, Catalytic
## 27169 D017412 0.036683786      Ribonucleoprotein, U1 Small Nuclear
## 40712 D019703 0.036683786                              Calcineurin
## 40721 D039601 0.036683786          Eukaryotic Initiation Factor-4A
## 40722 D049488 0.036683786 Phosphatidylethanolamine Binding Protein
## 40723 D054788 0.036683786           Ribosome Inactivating Proteins
## 5     D004251 0.043101944                DNA Transposable Elements
## 27170 D017931 0.047453802                              DNA Primers
```

```
# Store list of terms
headingListD <- summary(meshR)[!duplicated(summary(meshR)[,7]),c(7)]
```

Switching to a different category is easily done by the ‘category<-’ function. Here, we use **Diseases** (category = “C”).

```
category(meshParams) <- "C"
meshR <- meshHyperGTest(meshParams)
```

```
## Warning in .meshHyperGTestInternal(p): None of MeSH Term is significant !
```

```
summary(meshR)[!duplicated(summary(meshR)[,7]),c(1,2,7)]
```

```
## [1] MESHID   Pvalue   MESHTERM
## <0 rows> (or 0-length row.names)
```

```
# Store list of terms
 headingListC <- summary(meshR)[!duplicated(summary(meshR)[,7]),c(7)]
```

MeSH ORA for **Anatomy** (category = “A”).

```
category(meshParams) <- "A"
meshR <- meshHyperGTest(meshParams)
summary(meshR)[!duplicated(summary(meshR)[,7]),c(1,2,7)]
```

```
##        MESHID      Pvalue             MESHTERM
## 27257 D052584 0.001343107                Xylem
## 27261 D052585 0.001343107               Phloem
## 27    D032461 0.031035939   Chromosomes, Plant
## 1     D006056 0.036683786      Golgi Apparatus
## 26    D022162 0.036683786 Cytoplasmic Vesicles
## 5     D012270 0.040751131            Ribosomes
## 27173 D035264 0.041738271              Flowers
```

```
# Store list of terms
headingListA <- summary(meshR)[!duplicated(summary(meshR)[,7]),c(7)]
```

MeSH ORA for **Phenomena and Processes** (category = “G”).

```
category(meshParams) <- "G"
meshR <- meshHyperGTest(meshParams)
summary(meshR)[!duplicated(summary(meshR)[,7]),c(1,2,7)]
```

```
##        MESHID      Pvalue                           MESHTERM
## 40720 D026801 0.005429013                            Synteny
## 27123 D012641 0.022743530                 Selection, Genetic
## 40902 D032461 0.031035939                 Chromosomes, Plant
## 27164 D014774 0.036683786                          Virulence
## 27165 D015966 0.036683786 Gene Expression Regulation, Fungal
## 40648 D017434 0.038166707        Protein Structure, Tertiary
## 27167 D017398 0.039975954               Alternative Splicing
## 1     D004251 0.043101944          DNA Transposable Elements
```

```
# Store list of terms
headingListG <- summary(meshR)[!duplicated(summary(meshR)[,7]),c(7)]
```

## 5. Output list of significant MeSH headers

```
ImprovementMeshList <- list(headingListA,headingListC,headingListD,headingListG)
save(ImprovementMeshList,file="ImprovementMeshList.Robj")
```

## 6. Session Information

```
sessionInfo()
```

```
## R version 3.3.0 (2016-05-03)
## Platform: x86_64-redhat-linux-gnu (64-bit)
## Running under: Fedora 23 (Workstation Edition)
## 
## locale:
##  [1] LC_CTYPE=en_US.UTF-8       LC_NUMERIC=C              
##  [3] LC_TIME=en_US.UTF-8        LC_COLLATE=en_US.UTF-8    
##  [5] LC_MONETARY=en_US.UTF-8    LC_MESSAGES=en_US.UTF-8   
##  [7] LC_PAPER=en_US.UTF-8       LC_NAME=C                 
##  [9] LC_ADDRESS=C               LC_TELEPHONE=C            
## [11] LC_MEASUREMENT=en_US.UTF-8 LC_IDENTIFICATION=C       
## 
## attached base packages:
##  [1] grid      stats4    parallel  stats     graphics  grDevices utils    
##  [8] datasets  methods   base     
## 
## other attached packages:
##  [1] MeSH.Zma.eg.db_1.6.0     meshr_1.8.0             
##  [3] MeSH.Syn.eg.db_1.6.0     MeSH.Bsu.168.eg.db_1.6.0
##  [5] MeSH.Aca.eg.db_1.6.0     MeSH.Hsa.eg.db_1.6.0    
##  [7] MeSH.PCR.db_1.6.0        MeSH.AOR.db_1.6.0       
##  [9] MeSH.db_1.6.0            MeSHDbi_1.8.0           
## [11] org.Hs.eg.db_3.3.0       cummeRbund_2.14.0       
## [13] Gviz_1.16.1              rtracklayer_1.32.1      
## [15] GenomicRanges_1.24.2     GenomeInfoDb_1.8.1      
## [17] fastcluster_1.1.20       reshape2_1.4.1          
## [19] ggplot2_2.1.0            fdrtool_1.2.15          
## [21] corrplot_0.77            GSEABase_1.34.0         
## [23] annotate_1.50.0          XML_3.98-1.4            
## [25] GO.db_3.3.0              RSQLite_1.0.0           
## [27] DBI_0.4-1                GOSemSim_1.30.2         
## [29] GOstats_2.38.1           graph_1.50.0            
## [31] Category_2.38.0          Matrix_1.2-6            
## [33] AnnotationDbi_1.34.4     IRanges_2.6.1           
## [35] S4Vectors_0.10.2         Biobase_2.32.0          
## [37] BiocGenerics_0.18.0      biomaRt_2.28.0          
## 
## loaded via a namespace (and not attached):
##  [1] bitops_1.0-6                  matrixStats_0.50.2           
##  [3] RColorBrewer_1.1-2            httr_1.2.1                   
##  [5] tools_3.3.0                   R6_2.1.2                     
##  [7] rpart_4.1-10                  Hmisc_3.17-4                 
##  [9] colorspace_1.2-6              nnet_7.3-12                  
## [11] gridExtra_2.2.1               chron_2.3-47                 
## [13] formatR_1.4                   scales_0.4.0                 
## [15] genefilter_1.54.2             RBGL_1.48.1                  
## [17] stringr_1.0.0                 digest_0.6.9                 
## [19] Rsamtools_1.24.0              foreign_0.8-66               
## [21] rmarkdown_1.0                 AnnotationForge_1.14.2       
## [23] XVector_0.12.0                dichromat_2.0-0              
## [25] htmltools_0.3.5               ensembldb_1.4.7              
## [27] BSgenome_1.40.1               BiocInstaller_1.22.3         
## [29] shiny_0.13.2                  BiocParallel_1.6.2           
## [31] acepack_1.3-3.3               VariantAnnotation_1.18.3     
## [33] RCurl_1.95-4.8                magrittr_1.5                 
## [35] Formula_1.2-1                 Rcpp_0.12.5                  
## [37] munsell_0.4.3                 stringi_1.1.1                
## [39] yaml_2.1.13                   SummarizedExperiment_1.2.3   
## [41] zlibbioc_1.18.0               plyr_1.8.4                   
## [43] AnnotationHub_2.4.2           lattice_0.20-33              
## [45] Biostrings_2.40.2             splines_3.3.0                
## [47] GenomicFeatures_1.24.4        knitr_1.13                   
## [49] evaluate_0.9                  biovizBase_1.20.0            
## [51] latticeExtra_0.6-28           data.table_1.9.6             
## [53] httpuv_1.3.3                  gtable_0.2.0                 
## [55] mime_0.5                      xtable_1.8-2                 
## [57] survival_2.39-5               GenomicAlignments_1.8.4      
## [59] cluster_2.0.4                 interactiveDisplayBase_1.10.3
```
