# Supplementary material for: Medical Subject Heading (MeSH) annotations illuminate maize genetics and evolution
Source: Plant Methods. 2017 Feb 23;13:8. doi: 10.1186/s13007-017-0159-5 (PMC5324291; doi:10.1186/s13007-017-0159-5)
Supplement: Supplementary file 3 — Additional file 3 R-Markdown file including script and results of MeSH and GO analysis on maize genes under selection for an increase in maize seed size. [file 13007_2017_159_MOESM3_ESM.html]

MeSH over-representation analysis (Maize Seed Size)


# MeSH over-representation analysis (Maize Seed Size)

## 0. Install BiomaRt

```
#source("https://bioconductor.org/biocLite.R")
#biocLite("biomaRt")
setwd("/home/beissinger/Documents/MESH_Maize/Manuscript/Supplemental Data/")
```

## 1. Create a vector of background genes

We first create a vector of background genes. We will use every gene with the required data (entrez id) as the background.

```
library(biomaRt)
## access to biomaRt
mart <- useMart(biomart = "plants_mart", host="plants.ensembl.org", dataset="zmays_eg_gene")
univ.geneID <- getBM(attributes=c("ensembl_gene_id", "entrezgene"), mart = mart) # 40481
## remove genes with no corresponding Entrez Gene ID
univ.geneID2 <- univ.geneID[!is.na(univ.geneID[,2]),] # 14142 
## remove duplicated Entrez Gene ID
univ.geneID3 <- univ.geneID2[ !duplicated(univ.geneID2[,2]),] # 13630
##Get GO terms
univ.geneID4<-getBM(attributes=c("entrezgene","go_accession","go_name_1006","go_namespace_1003","go_linkage_type"),mart=mart,filters='entrezgene',values=univ.geneID3$entrezgene)
## Code evidence for genes without GO terms as NA
univ.geneID5<-univ.geneID4[-which(univ.geneID4[,2]==""),]
###Make dataframe for GOStats
goframeData <- data.frame(go_id = univ.geneID5$go_accession, Evidence = univ.geneID5$go_linkage_type, gene_id = univ.geneID5$entrezgene,stringsAsFactors=F)
```

# 2. Create a vector of selected genes

Secondly, we create a vector of significant genes by reading the input file: Seed Size selected regions & identifying genes within these regions. Since these regions correspond to AGPv2, we use an archived gene build of AGPv2 to download appropriate data. Notice, I’ll investigate all regions, not only those from a particular divergence comparison.

```
## read data
allGenes<-read.table("ftp://ftp.gramene.org/pub/gramene/maizesequence.org/release-5b/filtered-set/ZmB73_5b_FGS_info.txt",header=T,stringsAsFactors=F)
allGenes$chromosome <- substr(allGenes$chromosome,4,6)
allGenes$chromosome <- as.numeric(allGenes$chromosome)
allGenes <- allGenes[which(is.na(allGenes$chromosome)==F),]
allGenes <- allGenes[!duplicated(allGenes$gene_id),] # only work with first transcript
my.regions <- read.table("/home/beissinger/Documents/MESH_Maize/Hirsch_Data/SeedSizeRegions.txt", header=T,stringsAsFactors = F)

### DOn't do the below, but leave it in as an option
#my.regions <- my.regions[which(my.regions$KLSKSS == 1),] ### KLS30 vs KSS30 regions
#my.regions <- my.regions[which(my.regions$KC0KLS == 1),] ### KLS30 vs KSS30 regions
#my.regions <- my.regions[which(my.regions$KC0KSS == 1),] ### KLS30 vs KSS30 regions

my.regions$Chromosome <- as.numeric(substr(my.regions$Chromosome,4,6))

genesWithin <- matrix(NA,nrow=0,ncol=2)
colnames(genesWithin)<- c("region","gene_id")
for(i in 1:nrow(my.regions)){
  #print(i)
  index <- which({allGenes$chromosome == my.regions$Chromosome[i] & allGenes$transcript_start >= my.regions$Start[i] & allGenes$transcript_start <= my.regions$End[i]} | 
          {allGenes$chromosome == my.regions$Chromosome[i] & allGenes$transcript_end >= my.regions$Start[i] & allGenes$transcript_end <= my.regions$End[i]} | 
          {allGenes$chromosome == my.regions$Chromosome[i] & allGenes$transcript_start <= my.regions$Start[i] & allGenes$transcript_end >= my.regions$End[i]})
  tmp<- cbind(as.numeric(rep(i,length(index))), allGenes$gene_id[index])
  genesWithin <- rbind(genesWithin, tmp)
}
my.geneID<-data.frame(genesWithin,stringsAsFactors=F)

colnames(my.geneID)[2] <- "ensembl_gene_id"
## merge two files
my.geneID2 <- merge(my.geneID, univ.geneID3, by ="ensembl_gene_id") # 898
## remove duplicated Entrez Gene ID
my.geneID3 <- my.geneID2[ !duplicated(my.geneID2$entrezgene),] # 862
```

## 3. GO enrichment analysis

We can perform a GO analysis using the *GOstats* package. Code to perform it is below.

```
#source("https://bioconductor.org/biocLite.R")
#biocLite("GOstats")
#biocLite("GOSemSim")
#biocLite("AnnotationForge")
#library("AnnotationForge")
#available.dbschemas() #maize is not available :-(
library("GOstats")
library("GOSemSim")
##Prepare GO to gene mappings
goFrame=GOFrame(goframeData,organism="Zea mays")
goAllFrame=GOAllFrame(goFrame)
library(GSEABase)
gsc <- GeneSetCollection(goAllFrame, setType = GOCollection())

params <- GSEAGOHyperGParams(name="Domestication Zea mays GO", geneSetCollection=gsc, geneIds = my.geneID3$entrezgene,
                             universeGeneIds = univ.geneID5$entrezgene, ontology = "BP", pvalueCutoff = 0.05, conditional = TRUE,
                             testDirection = "over")
```

GO enrichment analysis for **BP**

```
BP <- hyperGTest(params)
summary(BP)[,c(1,2,7)] # 28
```

```
##        GOBPID       Pvalue
## 1  GO:0055085 0.0002419613
## 2  GO:0045931 0.0002953509
## 3  GO:1901989 0.0002953509
## 4  GO:0007064 0.0002953509
## 5  GO:0006486 0.0004018186
## 6  GO:0009100 0.0004672312
## 7  GO:0007067 0.0005403794
## 8  GO:0010043 0.0008494819
## 9  GO:1901990 0.0014813050
## 10 GO:0070085 0.0015774851
## 11 GO:0032456 0.0026673579
## 12 GO:0033047 0.0029741605
## 13 GO:0051306 0.0029741605
## 14 GO:1902099 0.0029741605
## 15 GO:0051983 0.0029741605
## 16 GO:0010564 0.0030546973
## 17 GO:0034184 0.0044432586
## 18 GO:0051785 0.0044432586
## 19 GO:0048729 0.0044432586
## 20 GO:0048730 0.0044432586
## 21 GO:0060250 0.0044432586
## 22 GO:0045876 0.0044432586
## 23 GO:0045842 0.0044432586
## 24 GO:0034091 0.0044432586
## 25 GO:0080038 0.0044432586
## 26 GO:1902578 0.0058118702
## 27 GO:0006810 0.0067559563
## 28 GO:0010413 0.0068858737
## 29 GO:0015991 0.0068858737
## 30 GO:0000038 0.0084373116
## 31 GO:0006659 0.0084373116
## 32 GO:1902600 0.0100757468
## 33 GO:0045454 0.0123234080
## 34 GO:0009166 0.0127390742
## 35 GO:1903364 0.0127390742
## 36 GO:0032876 0.0127390742
## 37 GO:0045862 0.0127390742
## 38 GO:1901800 0.0127390742
## 39 GO:0032434 0.0183142533
## 40 GO:1903050 0.0183142533
## 41 GO:0090329 0.0187183523
## 42 GO:0031329 0.0187183523
## 43 GO:0045492 0.0226182263
## 44 GO:0080024 0.0243555322
## 45 GO:0016072 0.0253024251
## 46 GO:0034655 0.0281035592
## 47 GO:0006102 0.0325607396
## 48 GO:0044786 0.0355286926
## 49 GO:0044038 0.0355286926
## 50 GO:0043086 0.0357094104
## 51 GO:0010206 0.0388143152
## 52 GO:0008156 0.0388143152
## 53 GO:0000819 0.0406630448
## 54 GO:0006635 0.0427017836
## 55 GO:0045184 0.0457010869
## 56 GO:0010053 0.0475268492
## 57 GO:0016226 0.0475268492
## 58 GO:1902582 0.0482917711
## 59 GO:1901137 0.0483457996
##                                                                        Term
## 1                                                   transmembrane transport
## 2                                 positive regulation of mitotic cell cycle
## 3                        positive regulation of cell cycle phase transition
## 4                                         mitotic sister chromatid cohesion
## 5                                                     protein glycosylation
## 6                                            glycoprotein metabolic process
## 7                                                  mitotic nuclear division
## 8                                                      response to zinc ion
## 9                         regulation of mitotic cell cycle phase transition
## 10                                                            glycosylation
## 11                                                      endocytic recycling
## 12                       regulation of mitotic sister chromatid segregation
## 13                                      mitotic sister chromatid separation
## 14                regulation of metaphase/anaphase transition of cell cycle
## 15                                     regulation of chromosome segregation
## 16                                         regulation of cell cycle process
## 17  positive regulation of maintenance of mitotic sister chromatid cohesion
## 18                                  positive regulation of nuclear division
## 19                                                     tissue morphogenesis
## 20                                                  epidermis morphogenesis
## 21                                    germ-line stem-cell niche homeostasis
## 22                         positive regulation of sister chromatid cohesion
## 23             positive regulation of mitotic metaphase/anaphase transition
## 24                   regulation of maintenance of sister chromatid cohesion
## 25             positive regulation of cytokinin-activated signaling pathway
## 26                                             single-organism localization
## 27                                                                transport
## 28                                         glucuronoxylan metabolic process
## 29                                  ATP hydrolysis coupled proton transport
## 30                             very long-chain fatty acid metabolic process
## 31                                  phosphatidylserine biosynthetic process
## 32                                     hydrogen ion transmembrane transport
## 33                                                   cell redox homeostasis
## 34                                             nucleotide catabolic process
## 35                positive regulation of cellular protein catabolic process
## 36                             negative regulation of DNA endoreduplication
## 37                                       positive regulation of proteolysis
## 38             positive regulation of proteasomal protein catabolic process
## 39  regulation of proteasomal ubiquitin-dependent protein catabolic process
## 40 regulation of proteolysis involved in cellular protein catabolic process
## 41                              regulation of DNA-dependent DNA replication
## 42                                 regulation of cellular catabolic process
## 43                                               xylan biosynthetic process
## 44                                     indolebutyric acid metabolic process
## 45                                                   rRNA metabolic process
## 46                         nucleobase-containing compound catabolic process
## 47                                             isocitrate metabolic process
## 48                                               cell cycle DNA replication
## 49                             cell wall macromolecule biosynthetic process
## 50                                negative regulation of catalytic activity
## 51                                                    photosystem II repair
## 52                                   negative regulation of DNA replication
## 53                                             sister chromatid segregation
## 54                                                fatty acid beta-oxidation
## 55                                    establishment of protein localization
## 56                                      root epidermal cell differentiation
## 57                                             iron-sulfur cluster assembly
## 58                                  single-organism intracellular transport
## 59                             carbohydrate derivative biosynthetic process
```

```
# GO similarity
library(corrplot)
#goListBP <- summary(BP)[,c(1)]
#goSimMatBP <- mgoSim(goListBP, goListBP, ont="BP", measure="Wang", combine=NULL)
#corrplot(goSimMatBP, is.corr = FALSE, type="lower", tl.col = "black", tl.cex = 0.8)
```

GO enrichment analysis for **MF**

```
ontology(params) <- "MF"
MF <- hyperGTest(params)
summary(MF)[,c(1,2,7)] # 37
```

```
##        GOMFID      Pvalue
## 1  GO:0004449 0.002416994
## 2  GO:0005507 0.003732125
## 3  GO:0004637 0.004150266
## 4  GO:0009922 0.004150266
## 5  GO:0030145 0.004640168
## 6  GO:0004857 0.006599744
## 7  GO:0015035 0.008238627
## 8  GO:0019900 0.017415656
## 9  GO:0045735 0.020030956
## 10 GO:0017025 0.022819263
## 11 GO:0008649 0.022819263
## 12 GO:0045174 0.022819263
## 13 GO:0030599 0.022900807
## 14 GO:0050660 0.027521339
## 15 GO:0008762 0.029802347
## 16 GO:0004784 0.029802347
## 17 GO:0016668 0.029802347
## 18 GO:0015036 0.035720892
## 19 GO:0008378 0.046232702
##                                                                               Term
## 1                                         isocitrate dehydrogenase (NAD+) activity
## 2                                                               copper ion binding
## 3                                      phosphoribosylamine-glycine ligase activity
## 4                                                     fatty acid elongase activity
## 5                                                            manganese ion binding
## 6                                                        enzyme inhibitor activity
## 7                                        protein disulfide oxidoreductase activity
## 8                                                                   kinase binding
## 9                                                      nutrient reservoir activity
## 10                                                       TBP-class protein binding
## 11                                                 rRNA methyltransferase activity
## 12                                  glutathione dehydrogenase (ascorbate) activity
## 13                                                         pectinesterase activity
## 14                                             flavin adenine dinucleotide binding
## 15                                     UDP-N-acetylmuramate dehydrogenase activity
## 16                                                   superoxide dismutase activity
## 17 oxidoreductase activity, acting on a sulfur group of donors, NAD(P) as acceptor
## 18                                               disulfide oxidoreductase activity
## 19                                                  galactosyltransferase activity
```

```
# GO similarity
#goListMF <- summary(MF)[,c(1)]
#goSimMatMF <- mgoSim(goListMF, goListMF, ont="MF", measure="Wang", combine=NULL)
#corrplot(goSimMatMF, is.corr = FALSE, type="lower", tl.col = "black", tl.cex = 0.8)
```

GO enrichment analysis for **CC**

```
ontology(params) <- "CC"
CC <- hyperGTest(params)
summary(CC)[,c(1,2,7)] # 15
```

```
##        GOCCID       Pvalue                                          Term
## 1  GO:0033179 8.551259e-05  proton-transporting V-type ATPase, V0 domain
## 2  GO:0048046 2.186661e-03                                      apoplast
## 3  GO:0030915 7.969324e-03                             Smc5-Smc6 complex
## 4  GO:0016469 1.205501e-02 proton-transporting two-sector ATPase complex
## 5  GO:0030173 1.224527e-02          integral component of Golgi membrane
## 6  GO:0005773 1.933781e-02                                       vacuole
## 7  GO:0000326 2.343365e-02                       protein storage vacuole
## 8  GO:0031090 2.343875e-02                            organelle membrane
## 9  GO:0005740 2.480062e-02                        mitochondrial envelope
## 10 GO:0016020 2.799878e-02                                      membrane
## 11 GO:0005794 2.885150e-02                               Golgi apparatus
## 12 GO:0005741 3.187778e-02                  mitochondrial outer membrane
## 13 GO:0031977 3.503086e-02                               thylakoid lumen
## 14 GO:0030135 3.770359e-02                                coated vesicle
## 15 GO:0016021 4.962638e-02                integral component of membrane
```

```
# GO similarity
#goListCC <- summary(CC)[,c(1)]
#goSimMatCC <- mgoSim(goListCC, goListCC, ont="CC", measure="Wang", combine=NULL)
#corrplot(goSimMatCC[-4,-4], is.corr = FALSE, type="lower", tl.col = "black", tl.cex = 0.8)
```

## 4. MeSH enrichment analysis

Then, we perform a MeSH ORA for the category **Chemicals and Drugs** by setting ‘category=“D”’. Different categories are set as different letters, as will become clear in the following sections.

```
#biocLite("meshr")
#biocLite("MeSH.db")
#biocLite("MeSH.Zma.eg.db")
#biocLite("MeSHSim")
library(meshr)
library(MeSH.db)
library("MeSH.Zma.eg.db")
meshParams <- new("MeSHHyperGParams", geneIds = my.geneID3$entrezgene, universeGeneIds = univ.geneID3[,2], 
                  annotation = "MeSH.Zma.eg.db", category = "D", database = "gene2pubmed", 
                  pvalueCutoff = 0.05, pAdjust = "none")
meshR <- meshHyperGTest(meshParams)
summary(meshR)[!duplicated(summary(meshR)[,7]),c(1,2,7)]
```

```
##        MESHID      Pvalue                      MESHTERM
## 44796 D011061 0.003741985                        Poly A
## 1     D000644 0.003995311 Quaternary Ammonium Compounds
## 60591 D011422 0.003995311                   Propionates
## 60594 D014157 0.004317887         Transcription Factors
## 76625 D018076 0.005292734            DNA, Complementary
## 27239 D010940 0.006403371                Plant Proteins
## 60583 D011392 0.011481681                       Proline
## 11    D004251 0.017789964     DNA Transposable Elements
## 4     D002245 0.022002540                Carbon Dioxide
## 27133 D005287 0.035144991     Ferredoxin-NADP Reductase
## 27229 D010743 0.035144991                 Phospholipids
## 27142 D007527 0.040308922                    Isoenzymes
```

```
# Store list of terms
headingListD <- summary(meshR)[!duplicated(summary(meshR)[,7]),c(7)]
```

Switching to a different category is easily done by the ‘category<-’ function. Here, we use **Diseases** (category = “C”).

```
category(meshParams) <- "C"
meshR <- meshHyperGTest(meshParams)
```

```
## Warning in .meshHyperGTestInternal(p): None of MeSH Term is significant !
```

```
summary(meshR)[!duplicated(summary(meshR)[,7]),c(1,2,7)]
```

```
## [1] MESHID   Pvalue   MESHTERM
## <0 rows> (or 0-length row.names)
```

```
# Store list of terms
 headingListC <- summary(meshR)[!duplicated(summary(meshR)[,7]),c(7)]
```

MeSH ORA for **Anatomy** (category = “A”).

```
category(meshParams) <- "A"
meshR <- meshHyperGTest(meshParams)
summary(meshR)[!duplicated(summary(meshR)[,7]),c(1,2,7)]
```

```
##     MESHID     Pvalue           MESHTERM
## 1  D003600 0.01236630            Cytosol
## 91 D032461 0.01750824 Chromosomes, Plant
## 23 D018520 0.03347862       Plant Shoots
```

```
# Store list of terms
headingListA <- summary(meshR)[!duplicated(summary(meshR)[,7]),c(7)]
```

MeSH ORA for **Phenomena and Processes** (category = “G”).

```
category(meshParams) <- "G"
meshR <- meshHyperGTest(meshParams)
summary(meshR)[!duplicated(summary(meshR)[,7]),c(1,2,7)]
```

```
##         MESHID      Pvalue                                  MESHTERM
## 175393 D020641 0.002515776           Polymorphism, Single Nucleotide
## 145482 D020224 0.003379380                   Expressed Sequence Tags
## 129710 D018598 0.003457081                     Minisatellite Repeats
## 70973  D012689 0.006004691           Sequence Homology, Nucleic Acid
## 87830  D016678 0.011100571                                    Genome
## 1      D001483 0.012001678                             Base Sequence
## 87700  D015870 0.015654524                           Gene Expression
## 87811  D016296 0.015876726                               Mutagenesis
## 87842  D017343 0.017466983                              Genes, Plant
## 191727 D032461 0.017508238                        Chromosomes, Plant
## 43749  D004251 0.017789964                 DNA Transposable Elements
## 43727  D002940 0.021630234                          Circadian Rhythm
## 129703 D017385 0.022002540                         Sequence Homology
## 87649  D015854 0.028730825                             Up-Regulation
## 70924  D012150 0.034167686 Polymorphism, Restriction Fragment Length
## 87824  D016385 0.035144991                                  TATA Box
## 70871  D010766 0.038156266                           Phosphorylation
## 43702  D002455 0.044760196                             Cell Division
```

```
# Store list of terms
headingListG <- summary(meshR)[!duplicated(summary(meshR)[,7]),c(7)]
```

## 5. Output list of significant MeSH headers

```
SeedSizeMeshList <- list(headingListA,headingListC,headingListD,headingListG)
save(SeedSizeMeshList,file="SeedSizeMeshList.Robj")
```

## 6. Session Information

```
sessionInfo()
```

```
## R version 3.3.0 (2016-05-03)
## Platform: x86_64-redhat-linux-gnu (64-bit)
## Running under: Fedora 23 (Workstation Edition)
## 
## locale:
##  [1] LC_CTYPE=en_US.UTF-8       LC_NUMERIC=C              
##  [3] LC_TIME=en_US.UTF-8        LC_COLLATE=en_US.UTF-8    
##  [5] LC_MONETARY=en_US.UTF-8    LC_MESSAGES=en_US.UTF-8   
##  [7] LC_PAPER=en_US.UTF-8       LC_NAME=C                 
##  [9] LC_ADDRESS=C               LC_TELEPHONE=C            
## [11] LC_MEASUREMENT=en_US.UTF-8 LC_IDENTIFICATION=C       
## 
## attached base packages:
##  [1] grid      stats4    parallel  stats     graphics  grDevices utils    
##  [8] datasets  methods   base     
## 
## other attached packages:
##  [1] MeSH.Zma.eg.db_1.6.0     meshr_1.8.0             
##  [3] MeSH.Syn.eg.db_1.6.0     MeSH.Bsu.168.eg.db_1.6.0
##  [5] MeSH.Aca.eg.db_1.6.0     MeSH.Hsa.eg.db_1.6.0    
##  [7] MeSH.PCR.db_1.6.0        MeSH.AOR.db_1.6.0       
##  [9] MeSH.db_1.6.0            MeSHDbi_1.8.0           
## [11] org.Hs.eg.db_3.3.0       cummeRbund_2.14.0       
## [13] Gviz_1.16.1              rtracklayer_1.32.1      
## [15] GenomicRanges_1.24.2     GenomeInfoDb_1.8.1      
## [17] fastcluster_1.1.20       reshape2_1.4.1          
## [19] ggplot2_2.1.0            fdrtool_1.2.15          
## [21] corrplot_0.77            GSEABase_1.34.0         
## [23] annotate_1.50.0          XML_3.98-1.4            
## [25] GO.db_3.3.0              RSQLite_1.0.0           
## [27] DBI_0.4-1                GOSemSim_1.30.2         
## [29] GOstats_2.38.1           graph_1.50.0            
## [31] Category_2.38.0          Matrix_1.2-6            
## [33] AnnotationDbi_1.34.4     IRanges_2.6.1           
## [35] S4Vectors_0.10.2         Biobase_2.32.0          
## [37] BiocGenerics_0.18.0      biomaRt_2.28.0          
## 
## loaded via a namespace (and not attached):
##  [1] bitops_1.0-6                  matrixStats_0.50.2           
##  [3] RColorBrewer_1.1-2            httr_1.2.1                   
##  [5] tools_3.3.0                   R6_2.1.2                     
##  [7] rpart_4.1-10                  Hmisc_3.17-4                 
##  [9] colorspace_1.2-6              nnet_7.3-12                  
## [11] gridExtra_2.2.1               chron_2.3-47                 
## [13] formatR_1.4                   scales_0.4.0                 
## [15] genefilter_1.54.2             RBGL_1.48.1                  
## [17] stringr_1.0.0                 digest_0.6.9                 
## [19] Rsamtools_1.24.0              foreign_0.8-66               
## [21] rmarkdown_1.0                 AnnotationForge_1.14.2       
## [23] XVector_0.12.0                dichromat_2.0-0              
## [25] htmltools_0.3.5               ensembldb_1.4.7              
## [27] BSgenome_1.40.1               BiocInstaller_1.22.3         
## [29] shiny_0.13.2                  BiocParallel_1.6.2           
## [31] acepack_1.3-3.3               VariantAnnotation_1.18.3     
## [33] RCurl_1.95-4.8                magrittr_1.5                 
## [35] Formula_1.2-1                 Rcpp_0.12.5                  
## [37] munsell_0.4.3                 stringi_1.1.1                
## [39] yaml_2.1.13                   SummarizedExperiment_1.2.3   
## [41] zlibbioc_1.18.0               plyr_1.8.4                   
## [43] AnnotationHub_2.4.2           lattice_0.20-33              
## [45] Biostrings_2.40.2             splines_3.3.0                
## [47] GenomicFeatures_1.24.4        knitr_1.13                   
## [49] evaluate_0.9                  biovizBase_1.20.0            
## [51] latticeExtra_0.6-28           data.table_1.9.6             
## [53] httpuv_1.3.3                  gtable_0.2.0                 
## [55] mime_0.5                      xtable_1.8-2                 
## [57] survival_2.39-5               GenomicAlignments_1.8.4      
## [59] cluster_2.0.4                 interactiveDisplayBase_1.10.3
```
