# Supplementary material for: Medical Subject Heading (MeSH) annotations illuminate maize genetics and evolution
Source: Plant Methods. 2017 Feb 23;13:8. doi: 10.1186/s13007-017-0159-5 (PMC5324291; doi:10.1186/s13007-017-0159-5)
Supplement: Supplementary file 4 — Additional file 4 R-Markdown file including script and results of MeSH and GO analysis on maize genes under selection for an increase in ear number per plant. [file 13007_2017_159_MOESM4_ESM.html]

MeSH over-representation analysis (Maize Ear Number)


# MeSH over-representation analysis (Maize Ear Number)

## 0. Install BiomaRt

```
#source("https://bioconductor.org/biocLite.R")
#biocLite("biomaRt")
setwd("/home/beissinger/Documents/MESH_Maize/Manuscript/Supplemental Data/")
```

## 1. Create a vector of background genes

We first create a vector of background genes. We will use every gene with the required data (entrez id) as the background.

```
library(biomaRt)
## access to biomaRt
mart <- useMart(biomart = "plants_mart", host="plants.ensembl.org", dataset="zmays_eg_gene")
univ.geneID <- getBM(attributes=c("ensembl_gene_id", "entrezgene"), mart = mart) # 40481
## remove genes with no corresponding Entrez Gene ID
univ.geneID2 <- univ.geneID[!is.na(univ.geneID[,2]),] # 14142 
## remove duplicated Entrez Gene ID
univ.geneID3 <- univ.geneID2[ !duplicated(univ.geneID2[,2]),] # 13630
##Get GO terms
univ.geneID4<-getBM(attributes=c("entrezgene","go_accession","go_name_1006","go_namespace_1003","go_linkage_type"),mart=mart,filters='entrezgene',values=univ.geneID3$entrezgene)
## Code evidence for genes without GO terms as NA
univ.geneID5<-univ.geneID4[-which(univ.geneID4[,2]==""),]
###Make dataframe for GOStats
goframeData <- data.frame(go_id = univ.geneID5$go_accession, Evidence = univ.geneID5$go_linkage_type, gene_id = univ.geneID5$entrezgene,stringsAsFactors=F)
```

# 2. Create a vector of selected genes

Secondly, we create a vector of significant genes by reading the input file: Ear Number selected regions & identifying genes within these regions. Since these regions correspond to AGPv2, we use an archived gene build of AGPv2 to download appropriate data.

```
## read data
allGenes<-read.table("ftp://ftp.gramene.org/pub/gramene/maizesequence.org/release-5b/filtered-set/ZmB73_5b_FGS_info.txt",header=T,stringsAsFactors=F)
allGenes$chromosome <- substr(allGenes$chromosome,4,6)
allGenes$chromosome <- as.numeric(allGenes$chromosome)
allGenes <- allGenes[which(is.na(allGenes$chromosome)==F),]
allGenes <- allGenes[!duplicated(allGenes$gene_id),] # only work with first transcript
my.regions <- read.table("/home/beissinger/Documents/MESH_Maize/Beissinger_Data/EarNumberRegions.txt", header=T,stringsAsFactors = F)

genesWithin <- matrix(NA,nrow=0,ncol=2)
colnames(genesWithin)<- c("region","gene_id")
for(i in 1:nrow(my.regions)){
  #print(i)
  index <- which({allGenes$chromosome == my.regions$chrom[i] & allGenes$transcript_start >= my.regions$chromStart[i] & allGenes$transcript_start <= my.regions$chromEnd[i]} | 
          {allGenes$chromosome == my.regions$chrom[i] & allGenes$transcript_end >= my.regions$chromStart[i] & allGenes$transcript_end <= my.regions$chromEnd[i]} | 
          {allGenes$chromosome == my.regions$chrom[i] & allGenes$transcript_start <= my.regions$chromStart[i] & allGenes$transcript_end >= my.regions$chromEnd[i]})
  tmp<- cbind(as.numeric(rep(i,length(index))), allGenes$gene_id[index])
  genesWithin <- rbind(genesWithin, tmp)
}
my.geneID<-data.frame(genesWithin,stringsAsFactors=F)

colnames(my.geneID)[2] <- "ensembl_gene_id"
## merge two files
my.geneID2 <- merge(my.geneID, univ.geneID3, by ="ensembl_gene_id") # 62
## remove duplicated Entrez Gene ID
my.geneID3 <- my.geneID2[ !duplicated(my.geneID2$entrezgene),] # 62
```

## 3. GO enrichment analysis

We can perform a GO analysis using the *GOstats* package. Code to perform it is below.

```
#source("https://bioconductor.org/biocLite.R")
#biocLite("GOstats")
#biocLite("GOSemSim")
#biocLite("AnnotationForge")
#library("AnnotationForge")
#available.dbschemas() #maize is not available :-(
library("GOstats")
library("GOSemSim")
##Prepare GO to gene mappings
goFrame=GOFrame(goframeData,organism="Zea mays")
goAllFrame=GOAllFrame(goFrame)
library(GSEABase)
gsc <- GeneSetCollection(goAllFrame, setType = GOCollection())

params <- GSEAGOHyperGParams(name="Domestication Zea mays GO", geneSetCollection=gsc, geneIds = my.geneID3$entrezgene,
                             universeGeneIds = univ.geneID5$entrezgene, ontology = "BP", pvalueCutoff = 0.05, conditional = TRUE,
                             testDirection = "over")
```

GO enrichment analysis for **BP**

```
BP <- hyperGTest(params)
summary(BP)[,c(1,2,7)] # 28
```

```
##        GOBPID      Pvalue
## 1  GO:0031508 0.003721425
## 2  GO:0044237 0.004145961
## 3  GO:0006625 0.004425868
## 4  GO:0070828 0.007429493
## 5  GO:0044743 0.010012988
## 6  GO:0071806 0.010489593
## 7  GO:0007031 0.010489593
## 8  GO:0016560 0.011124252
## 9  GO:0071704 0.013044973
## 10 GO:0006228 0.014805747
## 11 GO:0032776 0.014805747
## 12 GO:0006183 0.014805747
## 13 GO:1902580 0.021988777
## 14 GO:0006796 0.022880993
## 15 GO:0006241 0.029400000
## 16 GO:0009148 0.029400000
## 17 GO:0009208 0.029400000
## 18 GO:0046939 0.032912089
## 19 GO:0044238 0.038667727
## 20 GO:1901068 0.040208650
## 21 GO:0006338 0.040208650
## 22 GO:0034645 0.042118173
## 23 GO:0034641 0.043601800
## 24 GO:0022406 0.043785671
## 25 GO:0043412 0.047050868
##                                                        Term
## 1                      pericentric heterochromatin assembly
## 2                                cellular metabolic process
## 3                           protein targeting to peroxisome
## 4                              heterochromatin organization
## 5                intracellular protein transmembrane import
## 6                           protein transmembrane transport
## 7                                   peroxisome organization
## 8            protein import into peroxisome matrix, docking
## 9                       organic substance metabolic process
## 10                                 UTP biosynthetic process
## 11                              DNA methylation on cytosine
## 12                                 GTP biosynthetic process
## 13                    single-organism cellular localization
## 14          phosphate-containing compound metabolic process
## 15                                 CTP biosynthetic process
## 16  pyrimidine nucleoside triphosphate biosynthetic process
## 17 pyrimidine ribonucleoside triphosphate metabolic process
## 18                               nucleotide phosphorylation
## 19                                primary metabolic process
## 20          guanosine-containing compound metabolic process
## 21                                     chromatin remodeling
## 22              cellular macromolecule biosynthetic process
## 23             cellular nitrogen compound metabolic process
## 24                                         membrane docking
## 25                               macromolecule modification
```

```
# GO similarity
library(corrplot)
#goListBP <- summary(BP)[,c(1)]
#goSimMatBP <- mgoSim(goListBP, goListBP, ont="BP", measure="Wang", combine=NULL)
#corrplot(goSimMatBP, is.corr = FALSE, type="lower", tl.col = "black", tl.cex = 0.8)
```

GO enrichment analysis for **MF**

```
ontology(params) <- "MF"
MF <- hyperGTest(params)
summary(MF)[,c(1,2,7)] # 37
```

```
##        GOMFID       Pvalue
## 1  GO:0004713 0.0001145969
## 2  GO:0030795 0.0042074141
## 3  GO:0004647 0.0042074141
## 4  GO:0003714 0.0125706335
## 5  GO:0048531 0.0125706335
## 6  GO:0016597 0.0127408657
## 7  GO:0010428 0.0167265755
## 8  GO:0010429 0.0167265755
## 9  GO:0004550 0.0167265755
## 10 GO:0004575 0.0167265755
## 11 GO:0008327 0.0167265755
## 12 GO:0052866 0.0167265755
## 13 GO:0016301 0.0196082120
## 14 GO:0010385 0.0208654966
## 15 GO:0004017 0.0208654966
## 16 GO:0033926 0.0249874646
## 17 GO:0043177 0.0312129726
## 18 GO:0042578 0.0405062149
##                                                     Term
## 1                       protein tyrosine kinase activity
## 2                 jasmonate O-methyltransferase activity
## 3                     phosphoserine phosphatase activity
## 4                     transcription corepressor activity
## 5                beta-1,3-galactosyltransferase activity
## 6                                     amino acid binding
## 7                                   methyl-CpNpG binding
## 8                                   methyl-CpNpN binding
## 9                 nucleoside diphosphate kinase activity
## 10                    sucrose alpha-glucosidase activity
## 11                                    methyl-CpG binding
## 12   phosphatidylinositol phosphate phosphatase activity
## 13                                       kinase activity
## 14                double-stranded methylated DNA binding
## 15                             adenylate kinase activity
## 16 glycopeptide alpha-N-acetylgalactosaminidase activity
## 17                                  organic acid binding
## 18                   phosphoric ester hydrolase activity
```

```
# GO similarity
#goListMF <- summary(MF)[,c(1)]
#goSimMatMF <- mgoSim(goListMF, goListMF, ont="MF", measure="Wang", combine=NULL)
#corrplot(goSimMatMF, is.corr = FALSE, type="lower", tl.col = "black", tl.cex = 0.8)
```

GO enrichment analysis for **CC**

```
ontology(params) <- "CC"
CC <- hyperGTest(params)
summary(CC)[,c(1,2,7)] # 15
```

```
##       GOCCID      Pvalue
## 1 GO:0010369 0.008275149
## 2 GO:0005778 0.040735626
## 3 GO:0033290 0.043395035
## 4 GO:0016282 0.043395035
## 5 GO:0044438 0.043395035
## 6 GO:0005852 0.046047503
##                                                 Term
## 1                                       chromocenter
## 2                               peroxisomal membrane
## 3               eukaryotic 48S preinitiation complex
## 4               eukaryotic 43S preinitiation complex
## 5                                     microbody part
## 6 eukaryotic translation initiation factor 3 complex
```

```
# GO similarity
#goListCC <- summary(CC)[,c(1)]
#goSimMatCC <- mgoSim(goListCC, goListCC, ont="CC", measure="Wang", combine=NULL)
#corrplot(goSimMatCC[-4,-4], is.corr = FALSE, type="lower", tl.col = "black", tl.cex = 0.8)
```

## 4. MeSH enrichment analysis

Then, we perform a MeSH ORA for the category **Chemicals and Drugs** by setting ‘category=“D”’. Different categories are set as different letters, as will become clear in the following sections.

```
#biocLite("meshr")
#biocLite("MeSH.db")
#biocLite("MeSH.Zma.eg.db")
#biocLite("MeSHSim")
library(meshr)
library(MeSH.db)
library("MeSH.Zma.eg.db")
meshParams <- new("MeSHHyperGParams", geneIds = my.geneID3$entrezgene, universeGeneIds = univ.geneID3[,2], 
                  annotation = "MeSH.Zma.eg.db", category = "D", database = "gene2pubmed", 
                  pvalueCutoff = 0.05, pAdjust = "none")
meshR <- meshHyperGTest(meshParams)
summary(meshR)[!duplicated(summary(meshR)[,7]),c(1,2,7)]
```

```
## [1] MESHID   Pvalue   MESHTERM
## <0 rows> (or 0-length row.names)
```

```
# Store list of terms
headingListD <- summary(meshR)[!duplicated(summary(meshR)[,7]),c(7)]
```

Switching to a different category is easily done by the ‘category<-’ function. Here, we use **Diseases** (category = “C”).

```
category(meshParams) <- "C"
meshR <- meshHyperGTest(meshParams)
```

```
## Error in data.frame(names(selected.mesh[i]), as.numeric(scores$p), as.numeric(scores$odds), : arguments imply differing number of rows: 0, 1
```

```
summary(meshR)[!duplicated(summary(meshR)[,7]),c(1,2,7)]
```

```
## [1] MESHID   Pvalue   MESHTERM
## <0 rows> (or 0-length row.names)
```

```
# Store list of terms
 headingListC <- summary(meshR)[!duplicated(summary(meshR)[,7]),c(7)]
```

MeSH ORA for **Anatomy** (category = “A”).

```
category(meshParams) <- "A"
meshR <- meshHyperGTest(meshParams)
summary(meshR)[!duplicated(summary(meshR)[,7]),c(1,2,7)]
```

```
##    MESHID     Pvalue  MESHTERM
## 1 D056625 0.04021365 Endosperm
```

```
# Store list of terms
headingListA <- summary(meshR)[!duplicated(summary(meshR)[,7]),c(7)]
```

MeSH ORA for **Phenomena and Processes** (category = “G”).

```
category(meshParams) <- "G"
meshR <- meshHyperGTest(meshParams)
summary(meshR)[!duplicated(summary(meshR)[,7]),c(1,2,7)]
```

```
##    MESHID     Pvalue         MESHTERM
## 1 D034622 0.03582525 RNA Interference
```

```
# Store list of terms
headingListG <- summary(meshR)[!duplicated(summary(meshR)[,7]),c(7)]
```

## 5. Output list of significant MeSH headers

```
EarNumberMeshList <- list(headingListA,headingListC,headingListD,headingListG)
save(EarNumberMeshList,file="EarNumberMeshList.Robj")
```

## 6. Session Information

```
sessionInfo()
```

```
## R version 3.3.0 (2016-05-03)
## Platform: x86_64-redhat-linux-gnu (64-bit)
## Running under: Fedora 23 (Workstation Edition)
## 
## locale:
##  [1] LC_CTYPE=en_US.UTF-8       LC_NUMERIC=C              
##  [3] LC_TIME=en_US.UTF-8        LC_COLLATE=en_US.UTF-8    
##  [5] LC_MONETARY=en_US.UTF-8    LC_MESSAGES=en_US.UTF-8   
##  [7] LC_PAPER=en_US.UTF-8       LC_NAME=C                 
##  [9] LC_ADDRESS=C               LC_TELEPHONE=C            
## [11] LC_MEASUREMENT=en_US.UTF-8 LC_IDENTIFICATION=C       
## 
## attached base packages:
##  [1] grid      stats4    parallel  stats     graphics  grDevices utils    
##  [8] datasets  methods   base     
## 
## other attached packages:
##  [1] MeSH.Zma.eg.db_1.6.0     meshr_1.8.0             
##  [3] MeSH.Syn.eg.db_1.6.0     MeSH.Bsu.168.eg.db_1.6.0
##  [5] MeSH.Aca.eg.db_1.6.0     MeSH.Hsa.eg.db_1.6.0    
##  [7] MeSH.PCR.db_1.6.0        MeSH.AOR.db_1.6.0       
##  [9] MeSH.db_1.6.0            MeSHDbi_1.8.0           
## [11] org.Hs.eg.db_3.3.0       cummeRbund_2.14.0       
## [13] Gviz_1.16.1              rtracklayer_1.32.1      
## [15] GenomicRanges_1.24.2     GenomeInfoDb_1.8.1      
## [17] fastcluster_1.1.20       reshape2_1.4.1          
## [19] ggplot2_2.1.0            fdrtool_1.2.15          
## [21] corrplot_0.77            GSEABase_1.34.0         
## [23] annotate_1.50.0          XML_3.98-1.4            
## [25] GO.db_3.3.0              RSQLite_1.0.0           
## [27] DBI_0.4-1                GOSemSim_1.30.2         
## [29] GOstats_2.38.1           graph_1.50.0            
## [31] Category_2.38.0          Matrix_1.2-6            
## [33] AnnotationDbi_1.34.4     IRanges_2.6.1           
## [35] S4Vectors_0.10.2         Biobase_2.32.0          
## [37] BiocGenerics_0.18.0      biomaRt_2.28.0          
## 
## loaded via a namespace (and not attached):
##  [1] bitops_1.0-6                  matrixStats_0.50.2           
##  [3] RColorBrewer_1.1-2            httr_1.2.1                   
##  [5] tools_3.3.0                   R6_2.1.2                     
##  [7] rpart_4.1-10                  Hmisc_3.17-4                 
##  [9] colorspace_1.2-6              nnet_7.3-12                  
## [11] gridExtra_2.2.1               chron_2.3-47                 
## [13] formatR_1.4                   scales_0.4.0                 
## [15] genefilter_1.54.2             RBGL_1.48.1                  
## [17] stringr_1.0.0                 digest_0.6.9                 
## [19] Rsamtools_1.24.0              foreign_0.8-66               
## [21] rmarkdown_1.0                 AnnotationForge_1.14.2       
## [23] XVector_0.12.0                dichromat_2.0-0              
## [25] htmltools_0.3.5               ensembldb_1.4.7              
## [27] BSgenome_1.40.1               BiocInstaller_1.22.3         
## [29] shiny_0.13.2                  BiocParallel_1.6.2           
## [31] acepack_1.3-3.3               VariantAnnotation_1.18.3     
## [33] RCurl_1.95-4.8                magrittr_1.5                 
## [35] Formula_1.2-1                 Rcpp_0.12.5                  
## [37] munsell_0.4.3                 stringi_1.1.1                
## [39] yaml_2.1.13                   SummarizedExperiment_1.2.3   
## [41] zlibbioc_1.18.0               plyr_1.8.4                   
## [43] AnnotationHub_2.4.2           lattice_0.20-33              
## [45] Biostrings_2.40.2             splines_3.3.0                
## [47] GenomicFeatures_1.24.4        knitr_1.13                   
## [49] evaluate_0.9                  biovizBase_1.20.0            
## [51] latticeExtra_0.6-28           data.table_1.9.6             
## [53] httpuv_1.3.3                  gtable_0.2.0                 
## [55] mime_0.5                      xtable_1.8-2                 
## [57] survival_2.39-5               GenomicAlignments_1.8.4      
## [59] cluster_2.0.4                 interactiveDisplayBase_1.10.3
```
