# Supplementary material for: Medical Subject Heading (MeSH) annotations illuminate maize genetics and evolution
Source: Plant Methods. 2017 Feb 23;13:8. doi: 10.1186/s13007-017-0159-5 (PMC5324291; doi:10.1186/s13007-017-0159-5)
Supplement: Supplementary file 5 — Additional file 5 R-Markdown file including script and results of MeSH and GO analysis on maize genes implicated in a GWAS study of maize inflorescence traits. [file 13007_2017_159_MOESM5_ESM.html]

MeSH over-representation analysis (Maize GWAS SNPs for inflorescence traits)


# MeSH over-representation analysis (Maize GWAS SNPs for inflorescence traits)

## 0. Install BiomaRt

```
#source("https://bioconductor.org/biocLite.R")
#biocLite("biomaRt")
setwd("/home/beissinger/Documents/MESH_Maize/Manuscript/Supplemental Data/")
```

## 1. Create a vector of background genes

We first create a vector of background genes. We will use every gene with the required data (entrez id) as the background.

```
library(biomaRt)
## access to biomaRt
mart <- useMart(biomart = "plants_mart", host="plants.ensembl.org", dataset="zmays_eg_gene")
univ.geneID <- getBM(attributes=c("ensembl_gene_id", "entrezgene"), mart = mart) # 40481
## remove genes with no corresponding Entrez Gene ID
univ.geneID2 <- univ.geneID[!is.na(univ.geneID[,2]),] # 14142 
## remove duplicated Entrez Gene ID
univ.geneID3 <- univ.geneID2[ !duplicated(univ.geneID2[,2]),] # 13630
##Get GO terms
univ.geneID4<-getBM(attributes=c("entrezgene","go_accession","go_name_1006","go_namespace_1003","go_linkage_type"),mart=mart,filters='entrezgene',values=univ.geneID3$entrezgene)
## Code evidence for genes without GO terms as NA
univ.geneID5<-univ.geneID4[-which(univ.geneID4[,2]==""),]
###Make dataframe for GOStats
goframeData <- data.frame(go_id = univ.geneID5$go_accession, Evidence = univ.geneID5$go_linkage_type, gene_id = univ.geneID5$entrezgene,stringsAsFactors=F)
```

# 2. Create a vector of selected genes

Secondly, we create a vector of significant genes by reading the input file: Inflorescence trait GWAS SNPs & identifying genes that these SNPs correspond to. Since these positions correspond to AGPv2, we use an archived gene build of AGPv2 to download appropriate data.

```
## read data
allGenes<-read.table("ftp://ftp.gramene.org/pub/gramene/maizesequence.org/release-5b/filtered-set/ZmB73_5b_FGS_info.txt",header=T,stringsAsFactors=F)
allGenes$chromosome <- substr(allGenes$chromosome,4,6)
allGenes$chromosome <- as.numeric(allGenes$chromosome)
allGenes <- allGenes[which(is.na(allGenes$chromosome)==F),]
allGenes <- allGenes[!duplicated(allGenes$gene_id),] # only work with first transcript

my.snps <- read.table("/home/beissinger/Documents/MESH_Maize/Brown_Data/journal.pgen.1002383.s008.csv", header=T,stringsAsFactors = F,sep=",",skip=1)

genesWithin <- matrix(NA,nrow=0,ncol=3)
colnames(genesWithin)<- c("snp","trait","gene_id")
for(i in 1:nrow(my.snps)){
  #print(i)
  index <- which({allGenes$chromosome == my.snps$chrom[i] & {allGenes$transcript_start-10000} <= my.snps$bp[i] & {allGenes$transcript_end + 10000} >= my.snps$bp[i]} ) # identify genes within 10kb of SNPs
  tmp<- cbind(as.numeric(rep(i,length(index))), rep(my.snps$trait[i],length(index)), allGenes$gene_id[index])
  genesWithin <- rbind(genesWithin, tmp)
}
my.geneID<-data.frame(genesWithin,stringsAsFactors=F)

colnames(my.geneID)[3] <- "ensembl_gene_id"
## merge two files
my.geneID2 <- merge(my.geneID, univ.geneID3, by ="ensembl_gene_id") # 476
## remove duplicated Entrez Gene ID
my.geneID3 <- my.geneID2[ !duplicated(my.geneID2$entrezgene),] # 391
```

## 3. GO enrichment analysis

We can perform a GO analysis using the *GOstats* package. Code to perform it is below.

```
#source("https://bioconductor.org/biocLite.R")
#biocLite("GOstats")
#biocLite("GOSemSim")
#biocLite("AnnotationForge")
#library("AnnotationForge")
#available.dbschemas() #maize is not available :-(
library("GOstats")
library("GOSemSim")
##Prepare GO to gene mappings
goFrame=GOFrame(goframeData,organism="Zea mays")
goAllFrame=GOAllFrame(goFrame)
library(GSEABase)
gsc <- GeneSetCollection(goAllFrame, setType = GOCollection())

params <- GSEAGOHyperGParams(name="Domestication Zea mays GO", geneSetCollection=gsc, geneIds = my.geneID3$entrezgene,
                             universeGeneIds = univ.geneID5$entrezgene, ontology = "BP", pvalueCutoff = 0.05, conditional = TRUE,
                             testDirection = "over")
```

GO enrichment analysis for **BP**

```
BP <- hyperGTest(params)
summary(BP)[,c(1,2,7)] # 71

# GO similarity
library(corrplot)
#goListBP <- summary(BP)[,c(1)]
#goSimMatBP <- mgoSim(goListBP, goListBP, ont="BP", measure="Wang", combine=NULL)
#corrplot(goSimMatBP, is.corr = FALSE, type="lower", tl.col = "black", tl.cex = 0.8)
```

GO enrichment analysis for **MF**

```
ontology(params) <- "MF"
MF <- hyperGTest(params)
summary(MF)[,c(1,2,7)] # 33
```

```
##        GOMFID       Pvalue
## 1  GO:0030246 0.0006221089
## 2  GO:0019789 0.0018388629
## 3  GO:0008113 0.0025225356
## 4  GO:0004590 0.0025225356
## 5  GO:0004588 0.0025225356
## 6  GO:0051861 0.0080863270
## 7  GO:0017089 0.0080863270
## 8  GO:0031418 0.0146970916
## 9  GO:0051015 0.0213608171
## 10 GO:0016229 0.0213608171
## 11 GO:1901682 0.0213608171
## 12 GO:0004448 0.0269385183
## 13 GO:0008184 0.0293381851
## 14 GO:0004370 0.0293381851
## 15 GO:0016413 0.0293381851
## 16 GO:0016972 0.0293381851
## 17 GO:0009917 0.0293381851
## 18 GO:0004489 0.0293381851
## 19 GO:0050342 0.0293381851
## 20 GO:0051670 0.0293381851
## 21 GO:2001070 0.0293381851
## 22 GO:0045552 0.0293381851
## 23 GO:0047911 0.0293381851
## 24 GO:0030366 0.0293381851
## 25 GO:0050213 0.0293381851
## 26 GO:0046428 0.0293381851
## 27 GO:0004844 0.0293381851
## 28 GO:0045486 0.0293381851
## 29 GO:0035299 0.0293381851
## 30 GO:0003865 0.0293381851
## 31 GO:0051213 0.0296919061
## 32 GO:0010181 0.0394625488
## 33 GO:0009982 0.0466204068
##                                                         Term
## 1                                       carbohydrate binding
## 2                                  SUMO transferase activity
## 3          peptide-methionine (S)-S-oxide reductase activity
## 4              orotidine-5'-phosphate decarboxylase activity
## 5                 orotate phosphoribosyltransferase activity
## 6                                         glycolipid binding
## 7                            glycolipid transporter activity
## 8                                    L-ascorbic acid binding
## 9                                     actin filament binding
## 10                            steroid dehydrogenase activity
## 11        sulfur compound transmembrane transporter activity
## 12                         isocitrate dehydrogenase activity
## 13                           glycogen phosphorylase activity
## 14                                  glycerol kinase activity
## 15                              O-acetyltransferase activity
## 16                                    thiol oxidase activity
## 17                         sterol 5-alpha reductase activity
## 18    methylenetetrahydrofolate reductase (NAD(P)H) activity
## 19                   tocopherol O-methyltransferase activity
## 20                                        inulinase activity
## 21                                            starch binding
## 22                    dihydrokaempferol 4-reductase activity
## 23             galacturan 1,4-alpha-galacturonidase activity
## 24                           molybdopterin synthase activity
## 25                   progesterone 5-alpha-reductase activity
## 26 1,4-dihydroxy-2-naphthoate octaprenyltransferase activity
## 27                         uracil DNA N-glycosylase activity
## 28                         naringenin 3-dioxygenase activity
## 29              inositol pentakisphosphate 2-kinase activity
## 30            3-oxo-5-alpha-steroid 4-dehydrogenase activity
## 31                                      dioxygenase activity
## 32                                               FMN binding
## 33                           pseudouridine synthase activity
```

```
# GO similarity
#goListMF <- summary(MF)[,c(1)]
#goSimMatMF <- mgoSim(goListMF, goListMF, ont="MF", measure="Wang", combine=NULL)
#corrplot(goSimMatMF, is.corr = FALSE, type="lower", tl.col = "black", tl.cex = 0.8)
```

GO enrichment analysis for **CC**

```
ontology(params) <- "CC"
CC <- hyperGTest(params)
summary(CC)[,c(1,2,7)] # 8
```

```
##       GOCCID      Pvalue                                 Term
## 1 GO:0005770 0.005473113                        late endosome
## 2 GO:0005794 0.012305622                      Golgi apparatus
## 3 GO:0009508 0.023537469                   plastid chromosome
## 4 GO:0032585 0.030909387         multivesicular body membrane
## 5 GO:0031429 0.030909387             box H/ACA snoRNP complex
## 6 GO:0032301 0.030909387                    MutSalpha complex
## 7 GO:0032302 0.030909387                     MutSbeta complex
## 8 GO:0031461 0.031305201 cullin-RING ubiquitin ligase complex
```

```
# GO similarity
#goListCC <- summary(CC)[,c(1)]
#goSimMatCC <- mgoSim(goListCC, goListCC, ont="CC", measure="Wang", combine=NULL)
#corrplot(goSimMatCC[-4,-4], is.corr = FALSE, type="lower", tl.col = "black", tl.cex = 0.8)
```

## 4. MeSH enrichment analysis

Then, we perform a MeSH ORA for the category **Chemicals and Drugs** by setting ‘category=“D”’.

```
#biocLite("meshr")
#biocLite("MeSH.db")
#biocLite("MeSH.Zma.eg.db")
#biocLite("MeSHSim")
library(meshr)
library(MeSH.db)
library("MeSH.Zma.eg.db")
meshParams <- new("MeSHHyperGParams", geneIds = my.geneID3$entrezgene, universeGeneIds = univ.geneID3[,2], 
                  annotation = "MeSH.Zma.eg.db", category = "D", database = "gene2pubmed", 
                  pvalueCutoff = 0.05, pAdjust = "none")
meshR <- meshHyperGTest(meshParams)
summary(meshR)[!duplicated(summary(meshR)[,7]),c(1,2,7)]
```

```
##     MESHID     Pvalue                                     MESHTERM
## 1  D004269 0.02585818                               DNA, Bacterial
## 17 D008034 0.02868672                                   Lincomycin
## 18 D010840 0.02868672                                 Phytosterols
## 19 D010938 0.02868672                                   Plant Oils
## 21 D011096 0.02868672                            Polygalacturonase
## 25 D011740 0.02868672                            Pyrimidine Dimers
## 27 D013741 0.02868672        3-Oxo-5-alpha-Steroid 4-Dehydrogenase
## 28 D018124 0.02868672             Receptors, Tumor Necrosis Factor
## 80 D042965 0.02868672 Methylenetetrahydrofolate Reductase (NADPH2)
## 82 D050684 0.02868672                    E2F Transcription Factors
## 84 D051398 0.02868672                                  Aquaporin 1
## 85 D051399 0.02868672                                  Aquaporin 2
## 29 D020033 0.03737889                             Protein Isoforms
```

```
# Store list of terms
headingListD <- summary(meshR)[!duplicated(summary(meshR)[,7]),c(7)]
```

Switching to a different category is easily done by the ‘category<-’ function. Here, we use **Diseases** (category = “C”).

```
category(meshParams) <- "C"
meshR <- meshHyperGTest(meshParams)
```

```
## Warning in .meshHyperGTestInternal(p): None of MeSH Term is significant !
```

```
summary(meshR)[!duplicated(summary(meshR)[,7]),c(1,2,7)]
```

```
## [1] MESHID   Pvalue   MESHTERM
## <0 rows> (or 0-length row.names)
```

```
# Store list of terms
 headingListC <- summary(meshR)[!duplicated(summary(meshR)[,7]),c(7)]
```

MeSH ORA for **Anatomy** (category = “A”).

```
category(meshParams) <- "A"
meshR <- meshHyperGTest(meshParams)
summary(meshR)[!duplicated(summary(meshR)[,7]),c(1,2,7)]
```

```
##      MESHID       Pvalue              MESHTERM
## 41  D004721 0.0002749431 Endoplasmic Reticulum
## 57  D018515 0.0118167061          Plant Leaves
## 1   D002462 0.0161344834         Cell Membrane
## 302 D020524 0.0380428990            Thylakoids
```

```
# Store list of terms
headingListA <- summary(meshR)[!duplicated(summary(meshR)[,7]),c(7)]
```

MeSH ORA for **Phenomena and Processes** (category = “G”).

```
category(meshParams) <- "G"
meshR <- meshHyperGTest(meshParams)
summary(meshR)[!duplicated(summary(meshR)[,7]),c(1,2,7)]
```

```
##       MESHID       Pvalue                          MESHTERM
## 18   D010641 0.0002552278                         Phenotype
## 4    D006823 0.0156668312                      Hybrid Vigor
## 186  D015870 0.0163101972                   Gene Expression
## 298  D018506 0.0192445751 Gene Expression Regulation, Plant
## 1376 D018533 0.0204963499                           Biomass
## 1    D004058 0.0286867205                         Diffusion
## 2    D004777 0.0286867205                       Environment
## 297  D018095 0.0286867205                Germ-Line Mutation
## 1436 D053843 0.0286867205               DNA Mismatch Repair
## 1438 D063246 0.0286867205              Organogenesis, Plant
## 1393 D020816 0.0448015904                 Amino Acid Motifs
```

```
# Store list of terms
headingListG <- summary(meshR)[!duplicated(summary(meshR)[,7]),c(7)]
```

## 5. Output list of significant MeSH headers

```
InflorescenceMeshList <- list(headingListA,headingListC,headingListD,headingListG)
save(InflorescenceMeshList,file="InflorescenceMeshList.Robj")
```

## 6. Session Information

```
sessionInfo()
```

```
## R version 3.3.0 (2016-05-03)
## Platform: x86_64-redhat-linux-gnu (64-bit)
## Running under: Fedora 23 (Workstation Edition)
## 
## locale:
##  [1] LC_CTYPE=en_US.UTF-8       LC_NUMERIC=C              
##  [3] LC_TIME=en_US.UTF-8        LC_COLLATE=en_US.UTF-8    
##  [5] LC_MONETARY=en_US.UTF-8    LC_MESSAGES=en_US.UTF-8   
##  [7] LC_PAPER=en_US.UTF-8       LC_NAME=C                 
##  [9] LC_ADDRESS=C               LC_TELEPHONE=C            
## [11] LC_MEASUREMENT=en_US.UTF-8 LC_IDENTIFICATION=C       
## 
## attached base packages:
##  [1] grid      stats4    parallel  stats     graphics  grDevices utils    
##  [8] datasets  methods   base     
## 
## other attached packages:
##  [1] MeSH.Zma.eg.db_1.6.0     meshr_1.8.0             
##  [3] MeSH.Syn.eg.db_1.6.0     MeSH.Bsu.168.eg.db_1.6.0
##  [5] MeSH.Aca.eg.db_1.6.0     MeSH.Hsa.eg.db_1.6.0    
##  [7] MeSH.PCR.db_1.6.0        MeSH.AOR.db_1.6.0       
##  [9] MeSH.db_1.6.0            MeSHDbi_1.8.0           
## [11] org.Hs.eg.db_3.3.0       cummeRbund_2.14.0       
## [13] Gviz_1.16.1              rtracklayer_1.32.1      
## [15] GenomicRanges_1.24.2     GenomeInfoDb_1.8.1      
## [17] fastcluster_1.1.20       reshape2_1.4.1          
## [19] ggplot2_2.1.0            fdrtool_1.2.15          
## [21] GSEABase_1.34.0          annotate_1.50.0         
## [23] XML_3.98-1.4             GO.db_3.3.0             
## [25] RSQLite_1.0.0            DBI_0.4-1               
## [27] GOSemSim_1.30.2          GOstats_2.38.1          
## [29] graph_1.50.0             Category_2.38.0         
## [31] Matrix_1.2-6             AnnotationDbi_1.34.4    
## [33] IRanges_2.6.1            S4Vectors_0.10.2        
## [35] Biobase_2.32.0           BiocGenerics_0.18.0     
## [37] biomaRt_2.28.0          
## 
## loaded via a namespace (and not attached):
##  [1] bitops_1.0-6                  matrixStats_0.50.2           
##  [3] RColorBrewer_1.1-2            httr_1.2.1                   
##  [5] tools_3.3.0                   R6_2.1.2                     
##  [7] rpart_4.1-10                  Hmisc_3.17-4                 
##  [9] colorspace_1.2-6              nnet_7.3-12                  
## [11] gridExtra_2.2.1               chron_2.3-47                 
## [13] formatR_1.4                   scales_0.4.0                 
## [15] genefilter_1.54.2             RBGL_1.48.1                  
## [17] stringr_1.0.0                 digest_0.6.9                 
## [19] Rsamtools_1.24.0              foreign_0.8-66               
## [21] rmarkdown_1.0                 AnnotationForge_1.14.2       
## [23] XVector_0.12.0                dichromat_2.0-0              
## [25] htmltools_0.3.5               ensembldb_1.4.7              
## [27] BSgenome_1.40.1               BiocInstaller_1.22.3         
## [29] shiny_0.13.2                  BiocParallel_1.6.2           
## [31] acepack_1.3-3.3               VariantAnnotation_1.18.3     
## [33] RCurl_1.95-4.8                magrittr_1.5                 
## [35] Formula_1.2-1                 Rcpp_0.12.5                  
## [37] munsell_0.4.3                 stringi_1.1.1                
## [39] yaml_2.1.13                   SummarizedExperiment_1.2.3   
## [41] zlibbioc_1.18.0               plyr_1.8.4                   
## [43] AnnotationHub_2.4.2           lattice_0.20-33              
## [45] Biostrings_2.40.2             splines_3.3.0                
## [47] GenomicFeatures_1.24.4        knitr_1.13                   
## [49] evaluate_0.9                  biovizBase_1.20.0            
## [51] latticeExtra_0.6-28           data.table_1.9.6             
## [53] httpuv_1.3.3                  gtable_0.2.0                 
## [55] mime_0.5                      xtable_1.8-2                 
## [57] survival_2.39-5               GenomicAlignments_1.8.4      
## [59] cluster_2.0.4                 interactiveDisplayBase_1.10.3
```
