# Supplementary material for: Medical Subject Heading (MeSH) annotations illuminate maize genetics and evolution
Source: Plant Methods. 2017 Feb 23;13:8. doi: 10.1186/s13007-017-0159-5 (PMC5324291; doi:10.1186/s13007-017-0159-5)
Supplement: Supplementary file 6 — Additional file 6 R-Markdown file including script and results of MeSH and GO analysis on a random set of 1500 maize genes. [file 13007_2017_159_MOESM6_ESM.html]

MeSH over-representation analysis (1500 randomly selected genes)


# MeSH over-representation analysis (1500 randomly selected genes)

## 0. Install BiomaRt and set seed (so results can be reproduced)

```
#source("https://bioconductor.org/biocLite.R")
#biocLite("biomaRt")
setwd("/home/beissinger/Documents/MESH_Maize/Manuscript/Supplemental Data/")
set.seed(651465) #I randomly hammered on my keyboard for this number. It makes the code reproducible, but can be changed for a different random  number generator.
```

## 1. Create a vector of background genes

We first create a vector of background genes. We will use every gene with the required data (entrez id) as the background.

```
library(biomaRt)
## access to biomaRt
mart <- useMart(biomart = "plants_mart", host="plants.ensembl.org", dataset="zmays_eg_gene")
univ.geneID <- getBM(attributes=c("ensembl_gene_id", "entrezgene"), mart = mart) # 40481
## remove genes with no corresponding Entrez Gene ID
univ.geneID2 <- univ.geneID[!is.na(univ.geneID[,2]),] # 14142 
## remove duplicated Entrez Gene ID
univ.geneID3 <- univ.geneID2[ !duplicated(univ.geneID2[,2]),] # 13630
##Get GO terms
univ.geneID4<-getBM(attributes=c("entrezgene","go_accession","go_name_1006","go_namespace_1003","go_linkage_type"),mart=mart,filters='entrezgene',values=univ.geneID3$entrezgene)
## Code evidence for genes without GO terms as NA
univ.geneID5<-univ.geneID4[-which(univ.geneID4[,2]==""),]
###Make dataframe for GOStats
goframeData <- data.frame(go_id = univ.geneID5$go_accession, Evidence = univ.geneID5$go_linkage_type, gene_id = univ.geneID5$entrezgene,stringsAsFactors=F)
```

# 2. Create a vector of selected genes

Secondly, we create a vector of significant genes by randomly choosing them! Since the other datasets were based on AGPv2, we again use an archived gene build of AGPv2 to download appropriate data.

```
## read data
allGenes<-read.table("ftp://ftp.gramene.org/pub/gramene/maizesequence.org/release-5b/filtered-set/ZmB73_5b_FGS_info.txt",header=T,stringsAsFactors=F)
allGenes$chromosome <- substr(allGenes$chromosome,4,6)
allGenes$chromosome <- as.numeric(allGenes$chromosome)
allGenes <- allGenes[which(is.na(allGenes$chromosome)==F),]
allGenes <- allGenes[!duplicated(allGenes$gene_id),] # only work with first transcript


my.geneID<-allGenes[sample(nrow(allGenes),1500),] #randomly sample genes

colnames(my.geneID)[2] <- "ensembl_gene_id"
## merge two files
my.geneID2 <- merge(my.geneID, univ.geneID3, by ="ensembl_gene_id")
## remove duplicated Entrez Gene ID
my.geneID3 <- my.geneID2[ !duplicated(my.geneID2$entrezgene),]
```

## 3. GO enrichment analysis

We perform a GO analysis using the *GOstats* package. This time we do refer to GO results in the manuscript, we the code below was run and results are part of this Rmarkdown file.

```
#source("https://bioconductor.org/biocLite.R")
#biocLite("GOstats")
#biocLite("GOSemSim")
#biocLite("AnnotationForge")
#library("AnnotationForge")
#available.dbschemas() #maize is not available :-(
library("GOstats")
library("GOSemSim")
##Prepare GO to gene mappings
goFrame=GOFrame(goframeData,organism="Zea mays")
goAllFrame=GOAllFrame(goFrame)
library(GSEABase)
gsc <- GeneSetCollection(goAllFrame, setType = GOCollection())

params <- GSEAGOHyperGParams(name="Domestication Zea mays GO", geneSetCollection=gsc, geneIds = my.geneID3$entrezgene,
                             universeGeneIds = univ.geneID5$entrezgene, ontology = "BP", pvalueCutoff = 0.05, conditional = TRUE,
                             testDirection = "over")
```

GO enrichment analysis for **BP**

```
BP <- hyperGTest(params)
summary(BP)[,c(1,2,7)] #37
```

```
##        GOBPID      Pvalue
## 1  GO:0002683 0.001440028
## 2  GO:0046470 0.005345651
## 3  GO:0006432 0.008211351
## 4  GO:0003333 0.011707286
## 5  GO:0016226 0.016963223
## 6  GO:0006820 0.017096016
## 7  GO:0016246 0.020381452
## 8  GO:0071705 0.024739080
## 9  GO:0031167 0.026643190
## 10 GO:0000724 0.029044180
## 11 GO:0009267 0.029424742
## 12 GO:0016441 0.031819309
## 13 GO:0009658 0.031848398
## 14 GO:0016559 0.034644233
## 15 GO:0006817 0.034644233
## 16 GO:0006835 0.034644233
## 17 GO:0006399 0.037857230
## 18 GO:0010342 0.038011696
## 19 GO:0008616 0.038011696
## 20 GO:0019433 0.038011696
## 21 GO:0009440 0.038011696
## 22 GO:0010529 0.038011696
## 23 GO:0046461 0.038011696
## 24 GO:0046473 0.038011696
## 25 GO:0045824 0.038011696
## 26 GO:0046503 0.038011696
## 27 GO:0098755 0.038011696
## 28 GO:0050687 0.038011696
## 29 GO:0010028 0.038011696
## 30 GO:0007143 0.038011696
## 31 GO:0032504 0.038664724
## 32 GO:0051567 0.038699873
## 33 GO:0010167 0.043442764
## 34 GO:0046942 0.045526522
##                                                       Term
## 1             negative regulation of immune system process
## 2                    phosphatidylcholine metabolic process
## 3                         phenylalanyl-tRNA aminoacylation
## 4                       amino acid transmembrane transport
## 5                             iron-sulfur cluster assembly
## 6                                          anion transport
## 7                                         RNA interference
## 8                              nitrogen compound transport
## 9                                         rRNA methylation
## 10 double-strand break repair via homologous recombination
## 11                         cellular response to starvation
## 12                      posttranscriptional gene silencing
## 13                                chloroplast organization
## 14                                      peroxisome fission
## 15                                 phosphate ion transport
## 16                             dicarboxylic acid transport
## 17                                  tRNA metabolic process
## 18                               endosperm cellularization
## 19                          queuosine biosynthetic process
## 20                          triglyceride catabolic process
## 21                               cyanate catabolic process
## 22                    negative regulation of transposition
## 23                         neutral lipid catabolic process
## 24                     phosphatidic acid metabolic process
## 25           negative regulation of innate immune response
## 26                          glycerolipid catabolic process
## 27            maintenance of seed dormancy by absisic acid
## 28        negative regulation of defense response to virus
## 29                                       xanthophyll cycle
## 30                                 female meiotic division
## 31                     multicellular organism reproduction
## 32                               histone H3-K9 methylation
## 33                                     response to nitrate
## 34                               carboxylic acid transport
```

```
# GO similarity
library(corrplot)
goListBP <- summary(BP)[,c(1)]
goSimMatBP <- mgoSim(goListBP, goListBP, ont="BP", measure="Wang", combine=NULL)
corrplot(goSimMatBP, is.corr = FALSE, type="lower", tl.col = "black", tl.cex = 0.8)
```

GO enrichment analysis for **MF**

```
ontology(params) <- "MF"
MF <- hyperGTest(params)
summary(MF)[,c(1,2,7)] # 37
```

```
##        GOMFID      Pvalue
## 1  GO:0003924 0.006905724
## 2  GO:0005315 0.008385183
## 3  GO:0004826 0.009186436
## 4  GO:0005342 0.010622745
## 5  GO:0015171 0.010850856
## 6  GO:0005515 0.014181980
## 7  GO:0000287 0.014807525
## 8  GO:0004630 0.014904810
## 9  GO:0070290 0.014904810
## 10 GO:0004185 0.016712881
## 11 GO:0005509 0.017750452
## 12 GO:0008289 0.021409188
## 13 GO:0016860 0.024621495
## 14 GO:0016817 0.027465902
## 15 GO:0016462 0.033238451
## 16 GO:0008514 0.038169926
## 17 GO:0001883 0.038459353
## 18 GO:0032555 0.038459353
## 19 GO:0005525 0.039958910
## 20 GO:0019001 0.039958910
## 21 GO:0008479 0.040254719
## 22 GO:0043715 0.040254719
## 23 GO:0043716 0.040254719
## 24 GO:0004314 0.040254719
## 25 GO:0016420 0.040254719
## 26 GO:0047627 0.040254719
## 27 GO:0046570 0.040254719
## 28 GO:0004485 0.040254719
## 29 GO:0008793 0.040254719
## 30 GO:0004165 0.040254719
## 31 GO:0004141 0.040254719
## 32 GO:0008824 0.040254719
## 33 GO:0000340 0.040254719
## 34 GO:0016885 0.040254719
## 35 GO:0010283 0.040254719
## 36 GO:0046480 0.040254719
## 37 GO:0046422 0.040254719
## 38 GO:0004073 0.040254719
## 39 GO:0004075 0.040254719
## 40 GO:0004015 0.040254719
## 41 GO:0032549 0.040711810
## 42 GO:0000049 0.048234356
##                                                                      Term
## 1                                                         GTPase activity
## 2                  inorganic phosphate transmembrane transporter activity
## 3                                      phenylalanine-tRNA ligase activity
## 4                         organic acid transmembrane transporter activity
## 5                           amino acid transmembrane transporter activity
## 6                                                         protein binding
## 7                                                   magnesium ion binding
## 8                                                phospholipase D activity
## 9        N-acylphosphatidylethanolamine-specific phospholipase D activity
## 10                                  serine-type carboxypeptidase activity
## 11                                                    calcium ion binding
## 12                                                          lipid binding
## 13                                 intramolecular oxidoreductase activity
## 14                          hydrolase activity, acting on acid anhydrides
## 15                                               pyrophosphatase activity
## 16                       organic anion transmembrane transporter activity
## 17                                              purine nucleoside binding
## 18                                          purine ribonucleotide binding
## 19                                                            GTP binding
## 20                                              guanyl nucleotide binding
## 21                               queuine tRNA-ribosyltransferase activity
## 22             2,3-diketo-5-methylthiopentyl-1-phosphate enolase activity
## 23 2-hydroxy-3-keto-5-methylthiopentenyl-1-phosphate phosphatase activity
## 24                   [acyl-carrier-protein] S-malonyltransferase activity
## 25                                            malonyltransferase activity
## 26                                             adenylylsulfatase activity
## 27                    methylthioribulose 1-phosphate dehydratase activity
## 28                               methylcrotonoyl-CoA carboxylase activity
## 29           aromatic-amino-acid:2-oxoglutarate aminotransferase activity
## 30                                dodecenoyl-CoA delta-isomerase activity
## 31                                         dethiobiotin synthase activity
## 32                                             cyanate hydratase activity
## 33                                      RNA 7-methylguanosine cap binding
## 34                           ligase activity, forming carbon-carbon bonds
## 35                                         pinoresinol reductase activity
## 36                            galactolipid galactosyltransferase activity
## 37                                     violaxanthin de-epoxidase activity
## 38                          aspartate-semialdehyde dehydrogenase activity
## 39                                            biotin carboxylase activity
## 40        adenosylmethionine-8-amino-7-oxononanoate transaminase activity
## 41                                                 ribonucleoside binding
## 42                                                           tRNA binding
```

```
# GO similarity
goListMF <- summary(MF)[,c(1)]
goSimMatMF <- mgoSim(goListMF, goListMF, ont="MF", measure="Wang", combine=NULL)
corrplot(goSimMatMF, is.corr = FALSE, type="lower", tl.col = "black", tl.cex = 0.8)
```

GO enrichment analysis for **CC**

```
ontology(params) <- "CC"
CC <- hyperGTest(params)
summary(CC)[,c(1,2,7)] # 15
```

```
##       GOCCID      Pvalue
## 1 GO:0000791 0.006847394
## 2 GO:0016272 0.016349128
## 3 GO:0042406 0.034651049
## 4 GO:0005720 0.034651049
## 5 GO:0005845 0.034651049
## 6 GO:0008540 0.036641031
## 7 GO:0005750 0.044773774
##                                                    Term
## 1                                           euchromatin
## 2                                     prefoldin complex
## 3 extrinsic component of endoplasmic reticulum membrane
## 4                               nuclear heterochromatin
## 5                              mRNA cap binding complex
## 6       proteasome regulatory particle, base subcomplex
## 7           mitochondrial respiratory chain complex III
```

```
# GO similarity
goListCC <- summary(CC)[,c(1)]
goSimMatCC <- mgoSim(goListCC, goListCC, ont="CC", measure="Wang", combine=NULL)
corrplot(goSimMatCC[-4,-4], is.corr = FALSE, type="lower", tl.col = "black", tl.cex = 0.8)
```

## 4. MeSH enrichment analysis

Then, we perform a MeSH ORA for the category **Chemicals and Drugs** by setting ‘category=“D”’. Different categories are set as different letters, as will become clear in the following sections.

```
#biocLite("meshr")
#biocLite("MeSH.db")
#biocLite("MeSH.Zma.eg.db")
#biocLite("MeSHSim")
library(meshr)
library(MeSH.db)
library("MeSH.Zma.eg.db")
meshParams <- new("MeSHHyperGParams", geneIds = my.geneID3$entrezgene, universeGeneIds = univ.geneID3[,2], 
                  annotation = "MeSH.Zma.eg.db", category = "D", database = "gene2pubmed", 
                  pvalueCutoff = 0.05, pAdjust = "none")
meshR <- meshHyperGTest(meshParams)
summary(meshR)[!duplicated(summary(meshR)[,7]),c(1,2,7)]
```

```
##     MESHID      Pvalue                                 MESHTERM
## 49 D010860 0.001402915                     Pigments, Biological
## 44 D010758 0.008004161                               Phosphorus
## 1  D002118 0.025992418                                  Calcium
## 17 D003545 0.033807299                                 Cysteine
## 34 D004255 0.037490829        Deoxyribodipyrimidine Photo-Lyase
## 41 D008034 0.037490829                               Lincomycin
## 42 D009569 0.037490829                             Nitric Oxide
## 55 D018808 0.037490829                Transcription Factor AP-1
## 62 D027425 0.037490829 Multidrug Resistance-Associated Proteins
## 65 D028061 0.037490829             Phosphate Transport Proteins
```

```
# Store list of terms
headingListD <- summary(meshR)[!duplicated(summary(meshR)[,7]),c(7)]
```

Switching to a different category is easily done by the ‘category<-’ function. Here, we use **Diseases** (category = “C”). For some reason, the **Diseases** category throws a strange error…

```
category(meshParams) <- "C"
meshR <- meshHyperGTest(meshParams)
```

```
## Warning in .meshHyperGTestInternal(p): None of MeSH Term is significant !
```

```
summary(meshR)[!duplicated(summary(meshR)[,7]),c(1,2,7)]
```

```
## [1] MESHID   Pvalue   MESHTERM
## <0 rows> (or 0-length row.names)
```

```
# Store list of terms
 headingListC <- summary(meshR)[!duplicated(summary(meshR)[,7]),c(7)]
```

MeSH ORA for **Anatomy** (category = “A”).

```
category(meshParams) <- "A"
meshR <- meshHyperGTest(meshParams)
summary(meshR)[!duplicated(summary(meshR)[,7]),c(1,2,7)]
```

```
##    MESHID       Pvalue           MESHTERM
## 4 D038821 9.476033e-06        Mycorrhizae
## 1 D008841 3.749083e-02 Actin Cytoskeleton
```

```
# Store list of terms
headingListA <- summary(meshR)[!duplicated(summary(meshR)[,7]),c(7)]
```

MeSH ORA for **Phenomena and Processes** (category = “G”).

```
category(meshParams) <- "G"
meshR <- meshHyperGTest(meshParams)
summary(meshR)[!duplicated(summary(meshR)[,7]),c(1,2,7)]
```

```
##     MESHID       Pvalue                     MESHTERM
## 13 D013559 2.758292e-05                    Symbiosis
## 1  D001693 4.103947e-03 Biological Transport, Active
## 59 D050260 3.380730e-02      Carbohydrate Metabolism
## 8  D004260 3.749083e-02                   DNA Repair
## 72 D053903 3.749083e-02  DNA Breaks, Double-Stranded
## 23 D016384 4.218623e-02           Consensus Sequence
```

```
# Store list of terms
headingListG <- summary(meshR)[!duplicated(summary(meshR)[,7]),c(7)]
```

## 5. Output list of significant MeSH headers

```
RandomMeshList <- list(headingListA,headingListC,headingListD,headingListG)
save(RandomMeshList,file="RandomMeshList.Robj")
```

## 6. Session Information

```
sessionInfo()
```

```
## R version 3.3.0 (2016-05-03)
## Platform: x86_64-redhat-linux-gnu (64-bit)
## Running under: Fedora 23 (Workstation Edition)
## 
## locale:
##  [1] LC_CTYPE=en_US.UTF-8       LC_NUMERIC=C              
##  [3] LC_TIME=en_US.UTF-8        LC_COLLATE=en_US.UTF-8    
##  [5] LC_MONETARY=en_US.UTF-8    LC_MESSAGES=en_US.UTF-8   
##  [7] LC_PAPER=en_US.UTF-8       LC_NAME=C                 
##  [9] LC_ADDRESS=C               LC_TELEPHONE=C            
## [11] LC_MEASUREMENT=en_US.UTF-8 LC_IDENTIFICATION=C       
## 
## attached base packages:
##  [1] grid      stats4    parallel  stats     graphics  grDevices utils    
##  [8] datasets  methods   base     
## 
## other attached packages:
##  [1] MeSH.Zma.eg.db_1.6.0     meshr_1.8.0             
##  [3] MeSH.Syn.eg.db_1.6.0     MeSH.Bsu.168.eg.db_1.6.0
##  [5] MeSH.Aca.eg.db_1.6.0     MeSH.Hsa.eg.db_1.6.0    
##  [7] MeSH.PCR.db_1.6.0        MeSH.AOR.db_1.6.0       
##  [9] MeSH.db_1.6.0            MeSHDbi_1.8.0           
## [11] org.Hs.eg.db_3.3.0       cummeRbund_2.14.0       
## [13] Gviz_1.16.1              rtracklayer_1.32.1      
## [15] GenomicRanges_1.24.2     GenomeInfoDb_1.8.1      
## [17] fastcluster_1.1.20       reshape2_1.4.1          
## [19] ggplot2_2.1.0            fdrtool_1.2.15          
## [21] corrplot_0.77            GSEABase_1.34.0         
## [23] annotate_1.50.0          XML_3.98-1.4            
## [25] GO.db_3.3.0              RSQLite_1.0.0           
## [27] DBI_0.4-1                GOSemSim_1.30.2         
## [29] GOstats_2.38.1           graph_1.50.0            
## [31] Category_2.38.0          Matrix_1.2-6            
## [33] AnnotationDbi_1.34.4     IRanges_2.6.1           
## [35] S4Vectors_0.10.2         Biobase_2.32.0          
## [37] BiocGenerics_0.18.0      biomaRt_2.28.0          
## 
## loaded via a namespace (and not attached):
##  [1] bitops_1.0-6                  matrixStats_0.50.2           
##  [3] RColorBrewer_1.1-2            httr_1.2.1                   
##  [5] tools_3.3.0                   R6_2.1.2                     
##  [7] rpart_4.1-10                  Hmisc_3.17-4                 
##  [9] colorspace_1.2-6              nnet_7.3-12                  
## [11] gridExtra_2.2.1               chron_2.3-47                 
## [13] formatR_1.4                   scales_0.4.0                 
## [15] genefilter_1.54.2             RBGL_1.48.1                  
## [17] stringr_1.0.0                 digest_0.6.9                 
## [19] Rsamtools_1.24.0              foreign_0.8-66               
## [21] rmarkdown_1.0                 AnnotationForge_1.14.2       
## [23] XVector_0.12.0                dichromat_2.0-0              
## [25] htmltools_0.3.5               ensembldb_1.4.7              
## [27] BSgenome_1.40.1               BiocInstaller_1.22.3         
## [29] shiny_0.13.2                  BiocParallel_1.6.2           
## [31] acepack_1.3-3.3               VariantAnnotation_1.18.3     
## [33] RCurl_1.95-4.8                magrittr_1.5                 
## [35] Formula_1.2-1                 Rcpp_0.12.5                  
## [37] munsell_0.4.3                 stringi_1.1.1                
## [39] yaml_2.1.13                   SummarizedExperiment_1.2.3   
## [41] zlibbioc_1.18.0               plyr_1.8.4                   
## [43] AnnotationHub_2.4.2           lattice_0.20-33              
## [45] Biostrings_2.40.2             splines_3.3.0                
## [47] GenomicFeatures_1.24.4        knitr_1.13                   
## [49] evaluate_0.9                  biovizBase_1.20.0            
## [51] latticeExtra_0.6-28           data.table_1.9.6             
## [53] httpuv_1.3.3                  gtable_0.2.0                 
## [55] mime_0.5                      xtable_1.8-2                 
## [57] survival_2.39-5               GenomicAlignments_1.8.4      
## [59] cluster_2.0.4                 interactiveDisplayBase_1.10.3
```
