# Supplementary material for: Medical Subject Heading (MeSH) annotations illuminate maize genetics and evolution
Source: Plant Methods. 2017 Feb 23;13:8. doi: 10.1186/s13007-017-0159-5 (PMC5324291; doi:10.1186/s13007-017-0159-5)
Supplement: Supplementary file 7 — Additional file 7 R-Markdown file including script and results of MeSH semantic similarity analysis. [file 13007_2017_159_MOESM7_ESM.html]

MeSH similarity analyisis (between all five datasets)


# MeSH similarity analyisis (between all five datasets)

## 0. Install BiomaRt and load the MeSHSim Package

```
#source("https://bioconductor.org/biocLite.R")
#biocLite("biomaRt")
setwd("/home/beissinger/Documents/MESH_Maize/Manuscript/Supplemental Data/")
library("MeSHSim")
```

## 1. Load the five files containing significant MeSH terms from each dataset

```
load("DomesticationMeshList.Robj")
load("ImprovementMeshList.Robj")
load("EarNumberMeshList.Robj")
load("SeedSizeMeshList.Robj")
load("InflorescenceMeshList.Robj")
load("RandomMeshList.Robj")
Domestication<-unlist(DomesticationMeshList)
Improvement<-unlist(ImprovementMeshList)
EarNumber<-unlist(EarNumberMeshList)
SeedSize<-unlist(SeedSizeMeshList)
Snps<-unlist(InflorescenceMeshList)
Random<-unlist(RandomMeshList)
```

## 1. Calculate similarity among MeSH terms from distinct datasets

```
similarityMat<-matrix(0,nrow=6,ncol=6) #create matrix to store values
rownames(similarityMat)<-c("Domestication","Improvement","Ear Number","Seed Size","Inflorescence", "Random")
colnames(similarityMat)<-c("Domestication","Improvement","Ear Number","Seed Size","Inflorescence", "Random")
similarityMat[2,1] <- headingSetSim(Domestication,Improvement)
similarityMat[3,1] <- headingSetSim(Domestication,EarNumber)
similarityMat[4,1] <- headingSetSim(Domestication,SeedSize)
similarityMat[5,1] <- headingSetSim(Domestication,Snps)
similarityMat[6,1] <- headingSetSim(Domestication,Random)

similarityMat[3,2] <- headingSetSim(Improvement,EarNumber)
similarityMat[4,2] <- headingSetSim(Improvement,SeedSize)
similarityMat[5,2] <- headingSetSim(Improvement,Snps)
similarityMat[6,2] <- headingSetSim(Improvement,Random)

similarityMat[4,3] <- headingSetSim(EarNumber,SeedSize)
similarityMat[5,3] <- headingSetSim(EarNumber,Snps)
similarityMat[6,3] <- headingSetSim(EarNumber,Random)

similarityMat[5,4] <- headingSetSim(SeedSize,Snps)
similarityMat[6,1] <- headingSetSim(SeedSize,Random)

similarityMat[6,5] <- headingSetSim(Snps,Random)
```

## 2. Make a plot

```
library(corrplot)
#pdf("SemanticSimilarity.pdf")
corrplot(similarityMat[1:5,1:5],method="circle",is.corr=FALSE,type="lower",tl.col="black",tl.cex=1,diag=F,tl.srt=45,addCoef.col="black",col=colorRampPalette(c("blue","white","red"))(200),cl.lim=c(0,1))
```

```
#dev.off()

#pdf("SemanticSimilarity_Full.pdf")
corrplot(similarityMat[1:6,1:6],method="circle",is.corr=FALSE,type="lower",tl.col="black",tl.cex=1,diag=F,tl.srt=45,addCoef.col="black",col=colorRampPalette(c("blue","white","red"))(200),cl.lim=c(0,1))
```

```
#dev.off()
```

## 5. Session Information

```
sessionInfo()
```

```
## R version 3.3.0 (2016-05-03)
## Platform: x86_64-redhat-linux-gnu (64-bit)
## Running under: Fedora 23 (Workstation Edition)
## 
## locale:
##  [1] LC_CTYPE=en_US.UTF-8       LC_NUMERIC=C              
##  [3] LC_TIME=en_US.UTF-8        LC_COLLATE=en_US.UTF-8    
##  [5] LC_MONETARY=en_US.UTF-8    LC_MESSAGES=en_US.UTF-8   
##  [7] LC_PAPER=en_US.UTF-8       LC_NAME=C                 
##  [9] LC_ADDRESS=C               LC_TELEPHONE=C            
## [11] LC_MEASUREMENT=en_US.UTF-8 LC_IDENTIFICATION=C       
## 
## attached base packages:
## [1] stats     graphics  grDevices utils     datasets  methods   base     
## 
## other attached packages:
## [1] corrplot_0.77 MeSHSim_1.4.0
## 
## loaded via a namespace (and not attached):
##  [1] magrittr_1.5    tools_3.3.0     htmltools_0.3.5 RCurl_1.95-4.8 
##  [5] yaml_2.1.13     Rcpp_0.12.5     stringi_1.1.1   rmarkdown_1.0  
##  [9] knitr_1.13      stringr_1.0.0   digest_0.6.9    bitops_1.0-6   
## [13] XML_3.98-1.4    evaluate_0.9
```
